# Supplementary material for: Perspectives on the origin of language: Infants vocalize most during independent vocal play but produce their most speech-like vocalizations during turn taking
Source: PLoS One. 2022 Dec 30;17(12):e0279395. doi: 10.1371/journal.pone.0279395 (PMC9803194; doi:10.1371/journal.pone.0279395)
Supplement: S1 Text — (PDF) [file pone.0279395.s001.pdf]

S1: On exclusion of sex and SES in the main analyses

To provide perspective, we tested for volubility and CBR in infants of high vs. low SES, and for boys vs. girls. There were no significant differences between the high and low SES groups for either volubility or CBR (unpaired t-test on volubility p = .45, on CBR p = .16), and there was no significant difference between boys and girls on CBR (p = .68). However, boys were more voluble than girls ($p < .01$), consistent with the results of a recent study from our laboratory that included the present dataset in addition to data on infants at risk for autism [1]. That study was also consistent with the present evaluation in that it reported no significant sex differences for CBR.

More importantly we tested for similarities between infants of high and low SES and between boys and girls on the testable propositions evaluated in the main text. Table A shows the data. The mean ratings for VP and TT were very similar to those found for the group as a whole as reported in the main text (N segments = 1572; mean VP = 3.61, SD = 1.26; mean TT = 1.24, SD = 0.53). T-tests revealed no significant differences except for boys vs girls on volubility, a variable not involved in the main text's testable propositions.

The four CBR figures show that for both boys and girls, and for both high and low SES, CBR was higher for Some TT and Some VP than for No TT and No VP. Similarly, the figures show that at both No TT and Some TT, CBRs were higher than for No VP and Some VP. In the case of Some TT and VP we interpret the difference as suggesting that infants produce their most speech-like forms when they are engaged in interaction. In the case of No VP and No TT, the difference is presumably explained primarily by the fact that segments with No TT were mostly included within the much larger set of segments with Some VP, and perhaps as well by higher

rates of crying, whimpering, and whining in the small number of segments with No VP than in the large number with No TT (see section F below).

**S1**	**N segments**	**Mean VP**	**SD VP**	**Mean TT**	**SD TT**
23 Boys	902	3.58	1.21	1.27	0.57
17 Girls	670	3.65	1.31	1.20	0.47
27 HiSES	1102	3.60	0.51	1.23	0.51
13 LoSES	470	3.62	0.57	1.26	0.57
**t-tests**	***df* = 38**	***p* level**			
Boys v Girls	CBR	0.679			
Boys v Girls	VP	0.743			
Boys v Girls	TT	0.219			
Boys v Girls	VOL	0.002**			
Hi v LoSES	CBR	0.159			
Hi v LoSES	VP	0.941			
Hi v LoSES	TT	0.654			
Hi v LoSES	VOL	0.453			

S1 Table. The data indicate that infants of high and low SES did not differ significantly on any of the variables addressed in the current study, nor even on Volubility (number of protophone utterances per minute). Boys and girls differed significantly (**) on Volubility, but not on any of the variables addressed in the current study.

Supporting Information

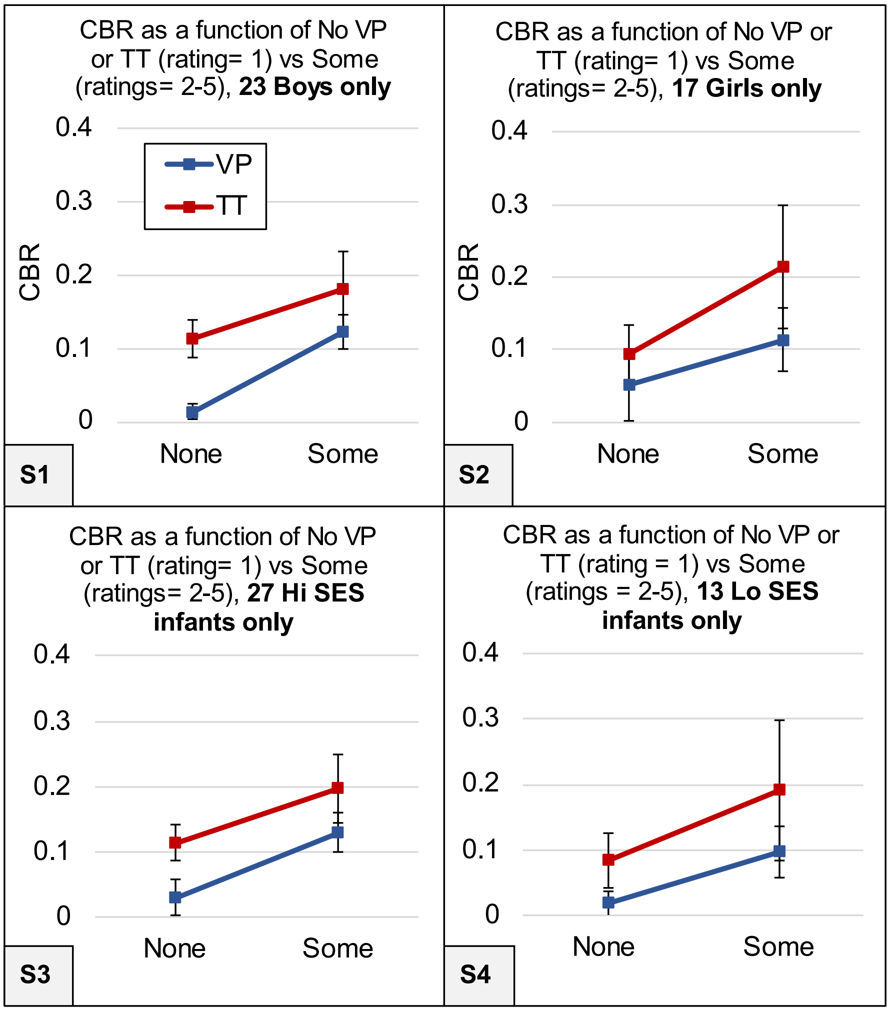

S1-S4 Figs: The figures show that the basic pattern of CBR findings (higher CBR at Some VP and Some TT than at No VP and No TT, as well as higher CBR at Some TT than at Some VP) applies to both boys and girls and to both high SES and Low SES. In addition, all 4 figures show higher CBR at No TT than No VP, but this fact is presumably largely due to the inclusion of the vast majority of segments with No TT (always a small number) in the much larger set of segments with Some VP, where CBRs are higher than at No VP.

References

1. Oller DK, Griebel U, Bowman DD, Bene ER, Long HL, Yoo H, et al. Infant boys found to be more vocal than infant girls. Current Biology. 2020;30(10): PR426-R7.
