# Supplementary material for: Perspectives on the origin of language: Infants vocalize most during independent vocal play but produce their most speech-like vocalizations during turn taking
Source: PLoS One. 2022 Dec 30;17(12):e0279395. doi: 10.1371/journal.pone.0279395 (PMC9803194; doi:10.1371/journal.pone.0279395)
Supplement: S2 Text — (PDF) [file pone.0279395.s002.pdf]

# S2: Abbreviated OLL coding instructions

The following descriptions of instructions to coders have been adjusted to use the same terminology as in the main text; the names of fields and codes used the OLL are not precisely the same as those presented here.

*For Phase 1*: In the Vocal Type Phonatory field, you will code infant “utterances” (one utterance per breath group), with focus on phonatory features (ignoring supraglottal articulation). Use only the following codes for protophones: Squeal, Vocant (vowel-like sound), Growl, Whisper, Ingress; for “fixed signals” use: Cry, Whimper, Laugh, and for protophones not fitting into the prior categories, use Other. For Repeat listening coding, select the codes from the AACT menu. For real-time coding, see keyboard shortcuts below:

| 1 | 2 | 3 | 4 | 5 | 6 | 7 | 8 | 9 | 0 |
|---|---|---|---|---|---|---|---|---|---|
| | Squeal | Vocant | Growl | Whisper | Ingress | Cry | Whimper | Laugh | Other |

***Definitions***

**Squeal:** High pitched (typically in falsetto or “loft”), out of range of the normal pitch of the infant

**Vocant:** Default category for protophones (neither squeal nor growl) typically with normal phonation, in the range of the normal pitch of the infant

**Growl:** Either a) low pitched, out of range of the normal pitch of the infant (typically in fry or “pulse”) or b) raucous, harsh voice (typically including subharmonics, biphonation, or phonatory chaos), not high pitched (i.e., growl can be either in the normal pitch range of the infant or below that range, but not above it)

**Supporting Information**

**Whisper:** Clearly audible sounds without the periodicity of phonation judged to be intentional as "vocalizations" (not merely the product of breathing). We code **Whisper** for totally voiceless utterances if they seem to be intentional vocalizations, potentially communicative

**Ingress:** Voiced ingressive sounds that are judged to be intentional as vocalizations (not merely the product of breathing). Do not code ingresses heard during cry bouts or incidentally occurring between protophones. Only code **Ingress** for isolated utterances with ingressive breath stream if they seem to be intentional vocalizations, potentially intended as communications. For ingressive-egressive sequences, code the whole sequence as one **Ingress**, as if it were one utterance (one breath group).

**Cry:** Wail (can also include glottal bursts or catch breaths or both, but the wail nucleus takes precedence in the judgment of Cry vs. Whimper); indicate one Cry for each breath group of Cry

**Whimper:** Glottal burst plus short, voiced nucleus, or whiny nucleus plus glottal burst (in general glottal bursts take precedence over any protophone features), indicate one Whimper for each breath group of Whimper

**Laugh:** This can consist of a single burst or multiple ones in a breath group (indicate one laugh for each breath group).

**Other:** Code **Other** for raspberries and consonant-like sounds occurring alone (e.g., **OthFric, Click, Burst**), but **NOT** for effort grunts or vegetative sounds (burp, sneeze, cough, etc.) unless you judge they are produced intentionally.

*For Phase 2*: In the Caregiver and Canonical Babbling field, you will code infant syllables as **CanSyl** or **NCSyl**; utterances where anyone talked to the infant will be coded as **AToIBRg** or **AToIAdRg**, and utterances where anyone talked to someone other than the infant as **AToANotI**. You will be coding both infant and other speakers simultaneously. For Repeat

listening coding, select the codes from the AACT menu. For real-time coding, see keyboard shortcuts below:

| 1 | 2 | 3 | 4 | 5 | 6 | 7 | 8 | 9 | 0 |
|---|---|---|---|---|---|---|---|---|---|
| | NCSyl | CanSyl | | | AToANotI | AToIBRg | AToIAdRg | | |

***Definitions***

**NCSyl**: non-canonical syllables within infant utterances (often just one in an utterance)

**CanSyl**: canonical syllables within infant utterances (often just one in an utterance)

**AToIBRg**: utterance where anyone talked to the baby in baby register

**AToIAdRg:** utterance where anyone talked to the baby in adult register

**AToANotI**: utterance where anyone talked to anyone, but not to the infant

For speakers other than the target infant, use intonation groups to code "utterances" rather than strict breath groups. Do not count more than one non-infant utterance if they are covering each other, i.e., if the non-infants are speaking simultaneously.

*Exclusion of utterances with low salience*

Utterances that are very low in intensity or very short may be irrelevant to possible communication and should not be coded at all. You have to make the judgment of whether you think an utterance is too low in salience based mostly on your own intuitions, but we will train on criterion setting. A particularly tricky case for such sounds is vocalizations occurring during suckling. In general, if the sounds seem to be intentional and potentially communicative, we code them in Phase 1 (usually as Vocant, but they can be Growl or Squeal as well).
