# Supplementary material for: Perspectives on the origin of language: Infants vocalize most during independent vocal play but produce their most speech-like vocalizations during turn taking
Source: PLoS One. 2022 Dec 30;17(12):e0279395. doi: 10.1371/journal.pone.0279395 (PMC9803194; doi:10.1371/journal.pone.0279395)
Supplement: S3 Text — (PDF) [file pone.0279395.s003.pdf]

S3: Questionnaire items for Phase 1 and Phase 2 coding

There are 34 questions in all, 17 for Phase 1 and 17 for Phase 2. Here we present only the questions that are involved in the analyses for the paper.

Background Information:

After you finish coding one session in the normal coding mode, you will be asked to fill out a Questionnaire. We have two types of Questionnaires: one for Phase 1 coding and another for Phase 2 coding. You will be asked to provide information about the circumstances of the recording (e.g., whether the baby was alone, asleep, interacting with another person), the baby's state (e.g., how often the baby was crying), the quality of the recording (e.g., how audible the infant vocalizations are, whether there was any radio or TV noise present), etc.

Questionnaire Protocol – Phase 1

Questions about infant vocal interaction across the session

1. Did any other person talk to the baby? This can be the parent or another adult or child.

On a scale of 1-5, rate how often another person talked to the infant (always in the Protocol, "the infant" refers to the "target" infant, or just "the baby", the one wearing the LENA recorder):
1 = Never, 2 = Less than half the time, 3 = About half the time, 4 = More than half the time, 5 = Close to the whole time. For example: Choose 1 if no one ever talked to the infant; choose 5 if another person talked to the infant the entire session, or close to the entire session.

Questionnaire Protocol – Phase 2

Questions about the functions of the protophones, if there were any protophones.

1. Were any of the infant's protophones used to complain? ***Include whining to try to get attention or to try to get an object, but do not include neutral or positive vocalizations. Do not include crying or whimpering because they are not protophones.***

Considering only the infant protophones, on a scale of 1-5, rate how many were used to try to get something (attention, an object, etc.) or merely to complain (including merely to express distress).

1 = None of them, 2 = Less than half of them, 3 = About half, 4 = More than half, 5 = All of them, n = No protophones to judge

3. Were any of the infant's protophones purely vocal play or vocal exploration (not social, not trying to get something, etc.)? *Fussing or complaining should not be counted also as vocal play or exploration, but vocal expressions of happiness or joy can be counted as instances of vocal play if they occur when the infant is not engaged in vocal interaction.*

On a scale of 1-5, rate how many of the protophones were used in play or exploration.
1 = None of them, 2 = Less than half of them, 3 = About half, 4 = More than half, 5 = All of them, n = No protophones to judge

4. Were any of the infant's protophones used in vocal turn-taking with another speaker? Do not include cases where a caregiver is talking to an infant who is crying but not producing protophones. Of course infant turn-taking can occur with utterances that are also expressions of joy or happiness, or even with expressions of complaint as long as they are not cry or whimper. So some protophones can serve functions of both complaint and turn-taking interaction, and others can serve functions of both exultation (expression of joy) and turn-taking interaction.

Considering only the infant protophones, on a scale of 1-5, rate how much of the session was vocal interaction with the protophones.
1= Never, 2 = Less than half the time, 3 = About half the time, 4 = More than half the time, 5 = Close to the whole time
