# Supplementary material for: Perspectives on the origin of language: Infants vocalize most during independent vocal play but produce their most speech-like vocalizations during turn taking
Source: PLoS One. 2022 Dec 30;17(12):e0279395. doi: 10.1371/journal.pone.0279395 (PMC9803194; doi:10.1371/journal.pone.0279395)
Supplement: S4 Text — (PDF) [file pone.0279395.s004.pdf]

S4: Additional Coder Agreement Data

The most important issue of coder agreement is whether the differences reported below in Results for 1572 segments can be confirmed to have been displayed in both the original coding of the 105 segments, and in the blind re-coding of the same 105 segments by different coders. The differences between levels of VP and TT assigned by the coders were stark in both the original coding and the blind recoding; paired t-tests showed that in both cases VP ratings were enormously higher than TT ratings ($p < .0001$, $d > 1.5$), with mean VP rating >3 in both cases, and mean TT rating < 1.3 in both cases.

For the same 7 coders with regard to original coding and re-coding, average CBR was almost identical (.0846 for original, .0845 for recoded). But the same did not occur for the 4 re-coders with respect to the original 15 (an average of .096 for the original, .133 for the coding by 4 new coders). The discrepancy for the pairings of different coders are not surprising, because coders working for many months on Ph1 and Ph2 coding tend to adopt somewhat different criteria for assigning canonical and non-canonical syllables, even though they are trained to a particular criterion on a standard set of training materials. Two of the 4 re-coders actually bracketed the original coder value (.096) quite closely (with CBRs of .108 and .091) while the other two produced higher values (.136 and .197).

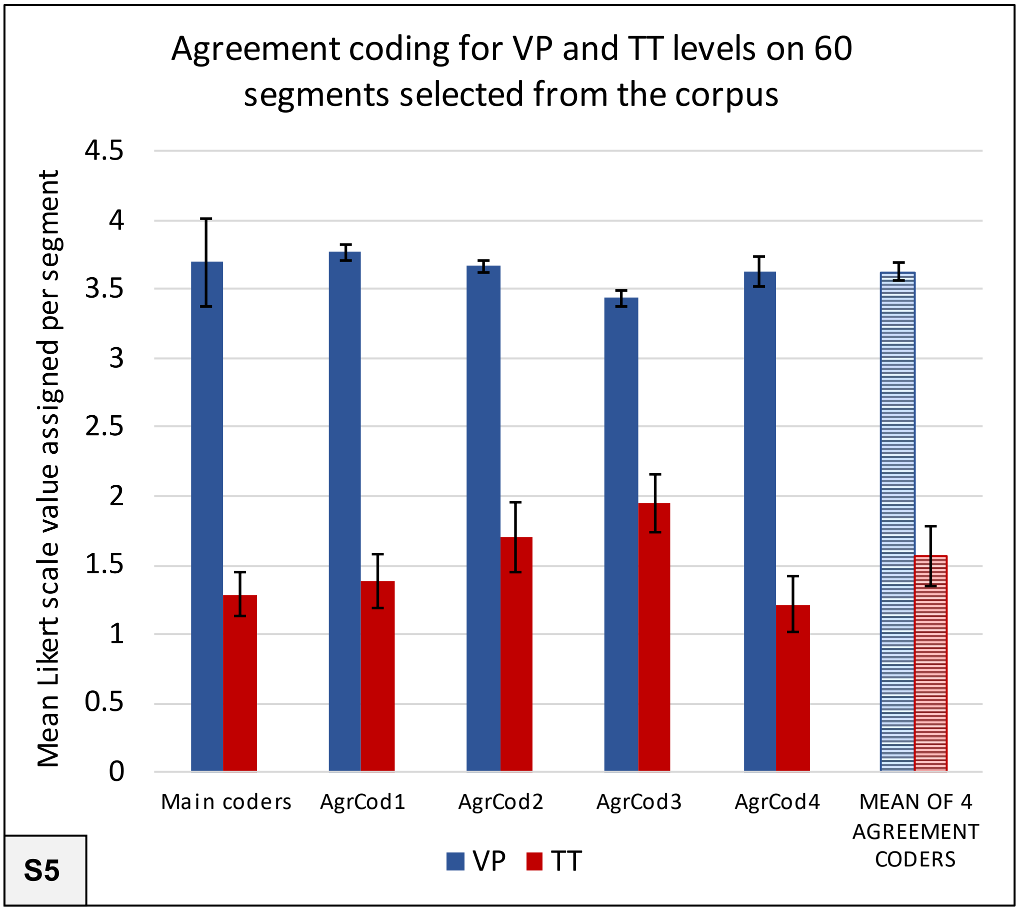

S5 Fig. 60 segments were selected from the 1572 to be re-coded, with blinding to the original codes, by 4 individuals who were available from the current coding group. Of particular interest was the question of whether different coders might have produced a different pattern of relation between amount of VP and amount of TT judged to have occurred in segments. There were notable differences among the four re-coders, with two of them (agreement coders 2 and 3) differing significantly (error bars are 95% confidence intervals) from the original (main) coders, but importantly all four re-coders agreed with the original coders in judging VP to have occurred vastly more often than TT with $p < .0001$ for all the re-coders as well the original coders.
