# Supplementary material for: Perspectives on the origin of language: Infants vocalize most during independent vocal play but produce their most speech-like vocalizations during turn taking
Source: PLoS One. 2022 Dec 30;17(12):e0279395. doi: 10.1371/journal.pone.0279395 (PMC9803194; doi:10.1371/journal.pone.0279395)
Supplement: S5 Text — (PDF) [file pone.0279395.s005.pdf]

S6: Additional data

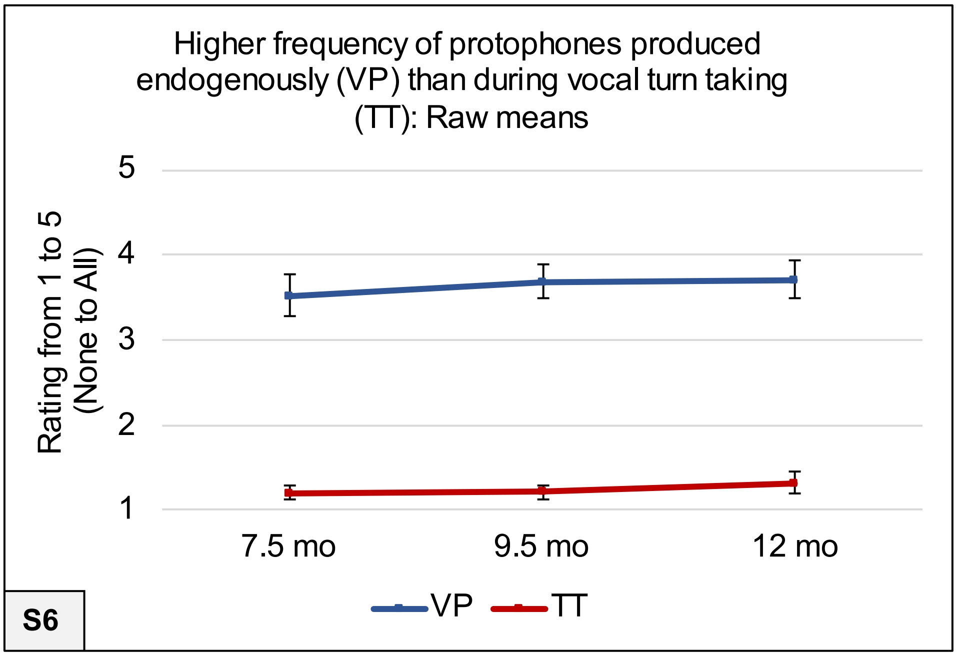

S6 Fig: In the main text, Figure 1A shows the differences between VP and TT based on ratings that were collapsed so that a rating of 1 was treated as None and a rating of 2 was treated as Some, that is, any rating of 2-5 was converted to 2. Figure S6 above shows the differences based on raw means and 95% CIs for VP vs TT ratings where the entire Likert Scale range is taken into account. The lowest possible rating was 1 and the highest 5. Thus, TT was deemed to have occurred very infrequently, with a mean rating only a little above 1, meaning TT occurred rarely. VP on the other hand showed a mean rating of over 3.5 at all three ages, indicating the coders judged VP to occur in most segments and most of the time during those segments.

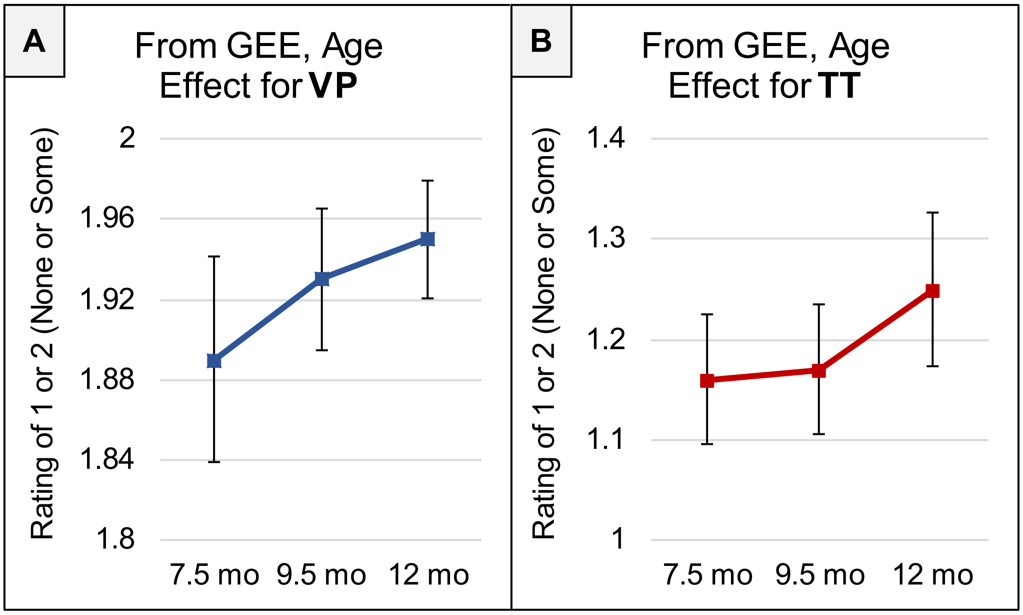

S7 Fig: The GEE analysis of VP (A) and TT (B) showed a small but significant effect of Age, paralleling the outcome seen in the raw data analysis of Figures 1C and 1D in the main text.

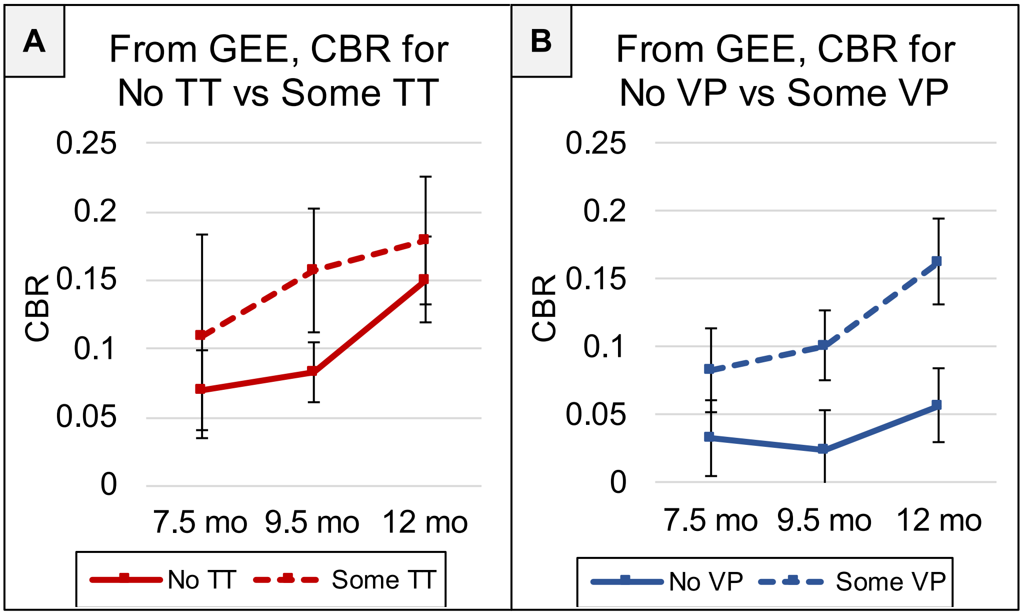

S8 Fig: GEE analysis confirmed the pattern observed in the raw data analysis displayed in Figure 2A and 2B of the main text, revealing a significantly higher CBR for Some VP and Some TT than for No VP and No TT. The GEE results appear, however, more conservative with regard to the differences than the raw data. This is a usual outcome with GEE analyses of data involving correlations across Age and missing cells. While the CBR effect size of the difference between

Some VP and No VP was small (*d* = .28) based on the GEE estimates, the effect as a percentage of the average CBR age difference was more impressive (96%). Similarly, the CBR effect size of the difference between Some TT and No TT was small (*d* = .14), but as a percentage of the average CBR age difference, the effect was substantial (58%).

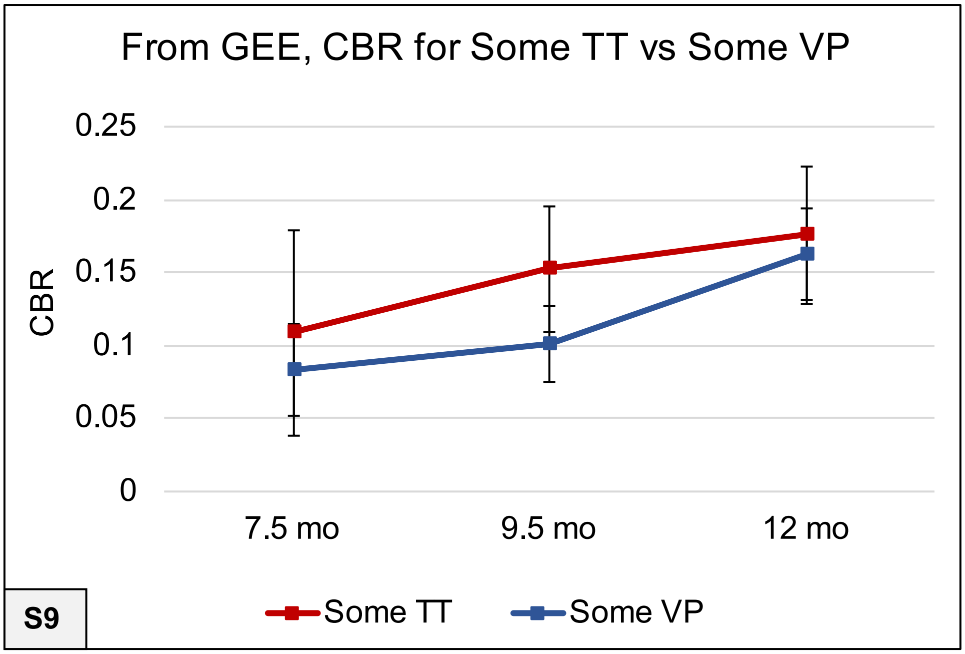

S9 Fig: Segments with Some TT showed significantly higher CBR than those with Some VP, although the average difference estimated by GEE across ages was small (*d* = .09), and once again the GEE results appear to be conservative with regard the extent of the differences seen in the raw data (Figure 2C of the main text). The size of the difference as indicated by the GEE estimates averaged across the three ages did, however, represent 37% of the range of CBR differences across the three ages.

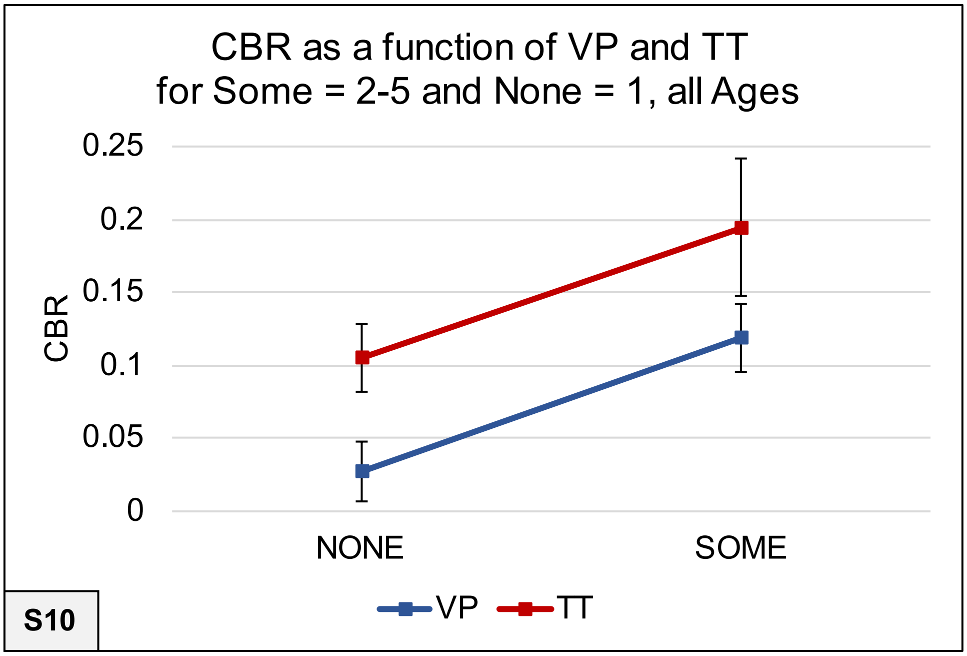

S10 Fig: Combining the data for None and Some for both VP and TT yields a picture that requires careful interpretation. Segments with No TT had higher CBR than those with No VP. The figure might at first glance be interpreted incorrectly. In fact, segments with No TT were, in the main, also segments that had Some VP, a condition that corresponds to relatively high CBR, as seen on the right side of the figure. An additional factor that may have contributed to low CBR in segments with No VP was negativity of infant vocalizations during the periods of No VP, see Figures S11 and S12.

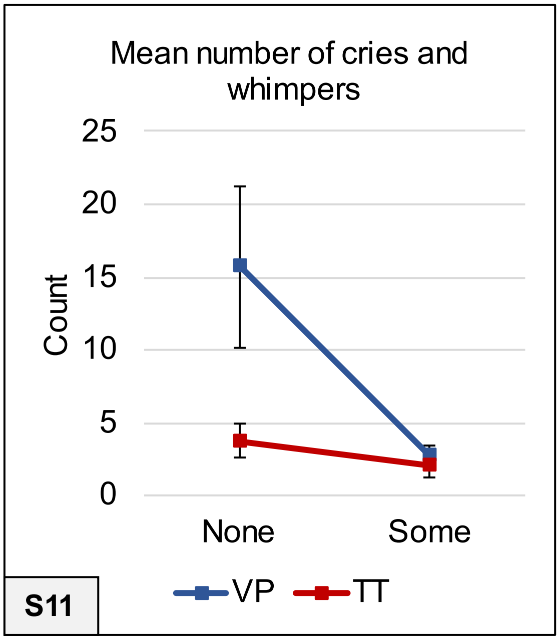

S11 Fig: Segments of recording where infants were not judged to be engaged in VP tended to be segments with more crying and whimpering than during segments where infants did engage in vocal play. We suppose that one of the reasons that CBR was very low in segments with No VP may have been that crying, and whimpering occupied a considerable amount of the time and that the infants may have been sufficiently uncomfortable that their inclination to produce VP was low.

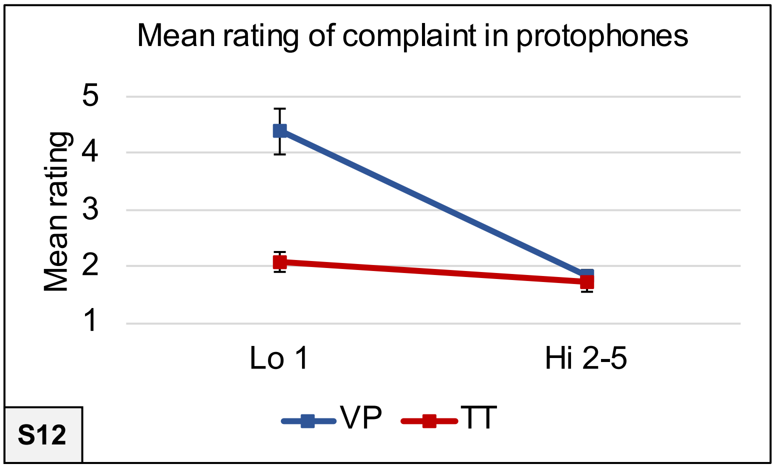

S12 Fig: Segments of recording where there was No VP showed high levels of complaint as indicated by the questionnaire item probing whine-like quality in the protophones of each 5-min segment. This was an independent judgment by the coders from crying and whimpering, which were coded, but were not counted as protophones. In accord with the coding instructions (see

above, section B), complaint sounds could not be treated as VP, and consequently it makes sense that high complaint ratings corresponded to low VP ratings. The high level of complaint at No VP may provide then another reason that CBR was very low at No VP.

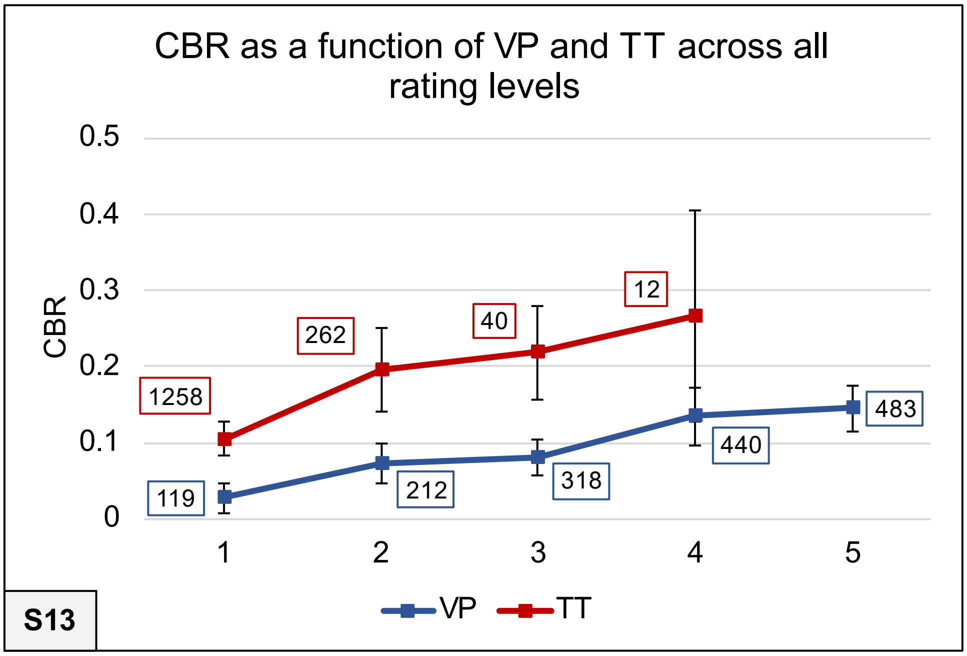

S13 Fig: Breakdown of the mean canonical babbling ratios (CBRs) for segments across each rating level (1-5) for VP and TT. All age data are collapsed within levels for both VP and TT. Data labels reflect the number of segments included in the mean calculation at each rating level. There were no segments that received a TT rating of 5 (i.e., TT occurring throughout the segment).
