# Supplementary material for: Perspectives on the origin of language: Infants vocalize most during independent vocal play but produce their most speech-like vocalizations during turn taking
Source: PLoS One. 2022 Dec 30;17(12):e0279395. doi: 10.1371/journal.pone.0279395 (PMC9803194; doi:10.1371/journal.pone.0279395)
Supplement: S6 Text — (PDF) [file pone.0279395.s006.pdf]

S7: Algorithm for Phase 2 Segment Selection

We select 8 segments from among the 21 that were randomly selected from a recording. We start by finding up to 4 that occur with potentially high interactivity while meeting other requirements.

1. Rank order the 21 segments according to volubility, i.e., the computed value for number of protophones from Ph1 coding [Squeal + Growl + Vocant].
2. Find up to 4 on this list (always selecting from the top down based on volubility) that meet the three criteria (eliminate all that do not meet each of the criteria) of a) high audibility (Ph1 question 10 or 11, <3), b) high enough IDS, i.e., IDS > 1, and c) high enough volubility, >5. Put those 4 or fewer in the basket for Ph2 as the “potentially interactive” segments.

At this point we will have selected at most 4 “potentially interactive” segments for Ph2. Now we seek at most 2 from among those remaining that are low in potential interactivity.

3. For each segment that is not already in the basket, add the IDS values on Ph1 question 1 to the reverse-ordered infant-alone values (Ph1, question 4) (i.e., transform the question 4 values this way: 5=1, 4=2, 3=3, 2=4, 1=5 and then add them to the IDS values). Maximum low-potential-interactivity will be treated as the lowest sum of these two (IDS plus reverse-ordered infant-alone).
4. From those segments that meet the criterion indicated in 3 (eliminate from contention all that do not meet the criterion) of high audibility (Ph1 question 10 or 11, <3), find the two lowest values on the sum of IDS and reverse-ordered infant-alone that have volubility > 5. These will be treated as the 2 that are low in potential interactivity (low IDS and high Alone) with the conditions that they have at least minimal volubility and high audibility. Pass these 2 (or fewer if there are <2 that meet the criteria) to the basket.

At this point we will have selected at most 6 segments for Ph2, but we need 8. So we choose the highest volubility segments that are still available to fill out the 8 for Ph2, that is, we choose at least the 2 with highest volubility, plus as many additional ones as necessary based on volubility to fill out the 8.

Supporting Information

5. From those not in the basket after anywhere from 0 – 4 have been passed to the basket based on IDS and volubility and anywhere from 0 – 2 have been passed to the basket based on low interactivity, start by seeking as many as necessary based on high volubility (selecting the 8th at random among ties for the 8th) until there are 8 in the basket, assuming they all meet the criterion of audibility (Ph1 question 10 or 11, <3). If you cannot fill out the 8 because of failure on the criterion of audibility, choose the last ones to fill out the 8 based on volubility alone.
6. Order the 8 selected for the final set for Ph2 by segment number (i.e., from morning to evening), pass this set to Ph2, and create the Ph2 coding assignment for the recording.
