# Supplementary material for: Perspectives on the origin of language: Infants vocalize most during independent vocal play but produce their most speech-like vocalizations during turn taking
Source: PLoS One. 2022 Dec 30;17(12):e0279395. doi: 10.1371/journal.pone.0279395 (PMC9803194; doi:10.1371/journal.pone.0279395)
Supplement: S1 Table — Amount of VP and TT by Likert-scale ratings by age. (PDF) [file pone.0279395.s007.pdf]

S5: Distributions of VP and TT by Age for all 5 Likert scale levels

A. Amount of VP and TT by Likert-scale Ratings for 7.5 mo

Number of segments

	None	Some			
**Rating**	**1**	**2**	**3**	**4**	**5**
**VP**	53	75	89	124	153
**TT**	413	69	8	4	0

Proportion of segments

	None	Some			
**Rating**	**1**	**2**	**3**	**4**	**5**
**VP**	0.11	0.15	0.18	0.25	0.31
**TT**	0.84	0.14	0.02	0.01	0.00

B. Amount of VP and TT by Likert-scale Ratings for 9.5 mo

Number of segments

	None	Some			
**Rating**	**1**	**2**	**3**	**4**	**5**
**VP**	33	54	96	152	159
**TT**	407	78	6	3	0

Proportion of segments

	None	Some			
**Rating**	**1**	**2**	**3**	**4**	**5**
**VP**	0.07	0.11	0.19	0.31	0.32
**TT**	0.82	0.16	0.01	0.01	0.00

C. Amount of VP and TT by Likert-scale Ratings for 12 mo

Number of segments

	None	Some			
**Rating**	**1**	**2**	**3**	**4**	**5**
**VP**	33	83	132	164	171
**TT**	438	114	26	5	0

Proportion of segments

	None	Some			
**Rating**	**1**	**2**	**3**	**4**	**5**
**VP**	0.06	0.14	0.23	0.28	0.29
**TT**	0.75	0.20	0.04	0.01	0.00

S5 Table: The imbalanced distribution of VP and TT applied to all three ages, such that TT was rated None for at least 75% of segments at all ages, while VP was rated None for not more than 11% of segments at any of the three ages.
