# Supplementary material for: Perspectives on the origin of language: Infants vocalize most during independent vocal play but produce their most speech-like vocalizations during turn taking
Source: PLoS One. 2022 Dec 30;17(12):e0279395. doi: 10.1371/journal.pone.0279395 (PMC9803194; doi:10.1371/journal.pone.0279395)
Supplement: Attachment — Submitted filename: Response to Reviewers.docx [file pone.0279395.s008.docx]

**All point-by-point responses to reviewers are bolded below**

PONE-D-22-03801

Canonical babbling during vocal turn taking and independent vocal play

PLOS ONE

Dear Dr. Long,

Thank you for submitting your manuscript to PLOS ONE. After careful consideration, we feel that it has merit but does not fully meet PLOS ONE’s publication criteria as it currently stands. Therefore, we invite you to submit a revised version of the manuscript that addresses the points raised during the review process.

I agree with Reviewer 2 that the abstract does not mention the two populations (children with LR vs HR for autism) and so seems to suggest a focus mainly on the drivers of canonical babbling. Please revise the abstract according to what the main emphasis of the study is, taking into account the comments below.

**The comments of the reviewers and the editor have helped us rethink our submission. In drafting the original submission, we included certain goals (those related autism and risk of autism) that we think now should have been left for a subsequent paper when data collection is complete in the broad NIDCD project from which the data are drawn. At the end of data collection, we will be able to address those goals more definitively. In writing the original submission as we did, we drew too much attention away from the two primary findings we think we should have focused on, namely:**

**A) the massive differences between the rates of occurrence of exploratory vocal play (here called VP) vs vocal turn taking (here called TT), and**

**B) the robust finding that canonical babbling ratio (CBR) is positively related within coded segments to amounts of both VP and TT, and the additional finding that TT seems to produce even higher CBR than VP.**

**The proper focus of the paper (and we have revised extensively to achieve this proper focus) should have addressed these two findings, A and B, for which the data are already conclusive. The less definitive findings related to interactions among risk, and the other factors should, we think, be left for a subsequent analysis and paper.**

**The revision also invokes the importance of exploratory vocal play to a greater extent than the original submission. The key idea is that the human infant appears to have been naturally selected to treat vocalization as an object of exploration and play in a way that parallels exploration and play with physical objects, namely toys or objects that can be treated as toys. This idea has been emphasized as a key to understanding why the human infant produces massive numbers of exploratory pre-speech sounds (protophones, ~3500 per day throughout the first year), while our ape relatives produce only comparatively miniscule numbers of sounds that can be thought to be analogous to protophones (Oller, Caskey, et al., 2019; Oller et al., 2021; Oller, Griebel, et al., 2019). It appears human infants are motivated internally to play with such sounds (~70-90% of protophones show no social directivity and no utilitarian purpose) but that other ape infants produce virtually all the sounds they can produce, not playfully, but with immediate utilitarian purposes (seeking attention, getting help, expressing fear or distress, etc.). These facts appear to demand an evolutionary explanation, which we and an independent colleague (John L. Locke) have argued to be founded in hominin altriciality, which we argue enhanced the natural selection pressure on fitness signaling by the human infant (Locke, 2006; Oller et al., 2016, 2021; Oller & Griebel, 2005). The vocal channel came under selection pressure to provide fitness information to caregivers in this way, according to our reasoning, and the result, after an uncertain number of generations, yielded a strong internal motivation by human infants (and mature humans for that matter) to find it pleasurable to explore vocal sound making. The only other proposed explanation that we know of for the massive amounts of vocalization by human infants is that it serves as practice and tuning of vocal skills for language. But that explanation can only be relevant once language exists. So is necessary still to seek an explanation for how vocal exploration came into being prior to language, and for that, the only proposed explanation on the table is based on the fitness signaling idea. The revised paper emphasizes the importance of taking account of the endogenous nature of human infant vocalization in the attempt to illuminate the origin of language—until vocal flexibility and high activity level in vocalization was present, further progress toward language was, we have argued, not possible, and it may similarly be impossible for the modern human infant to progress toward vocal language without exercising vocal flexibility in early infancy.**

**The focus on finding A in the revision thus provides new perspective on a fundamental issue in the development of precursors to speech in infancy (and on the evolution of language), an issue that has been portrayed incorrectly in the infancy literature for many decades. The focus of prior research on early language learning has been overwhelmingly on vocal interaction and turn-taking with caregivers portrayed as the source of vocal development in infants. But human coding of all-day recordings proves that the overwhelming tendency of infants is to produce protophones exploratorily rather than during turn-taking (i.e., during VP rather than TT). Consequently, the revision focuses heavily on this finding, portraying it prominently in the rationale and background, moving the key finding (previously in Table 3, in the Methods section) to the Results section under Proposition 1.**

**The focus on finding B in the revision (the relation between CBR and both VP and TT) is new information to the literature. We know of no prior research that has addressed CBR as a function of VP directly, and are aware of only indirect assessment of the possibility that TT is associated with higher CBR (Warlaumont et al., 2014). Yet these issues are of potentially fundamental importance both to the understanding of vocal development in general and to the proper formulation of intervention strategies in cases of abnormal vocal development. Perhaps the finding that will generate the most interest among those who have previously emphasized the importance of vocal interaction in infant development will be the greater CBR in segments with TT than in segments with VP. Both yield higher CBR than segments with neither VP nor TT, but segments with TT yielded the highest of all.**

**Because these two conclusive findings, A and B, apply to both HR and LR infants, and because the inclusion of a risk factor in the analysis introduces unnecessary complexity to the presentation while also requiring evaluation of issues that are not yet conclusive (as the reviewers noticed), we have resolved that the best way to revise is to eliminate the risk factor from the paper. Instead of two groups (high risk [HR] and low risk [LR]), we have revised to focus on only the 40 infants who were recruited as LR and who also received no clinical diagnosis in the most recent assessments. So instead of an n of 98, we now have an n of 40, which proves to provide plenty of power to illustrate the strong effects that are the focus of the revision. It is also the case that simplifying the study in this way makes it possible to also simplify the statistical analyses greatly.**

**The editor and reviewers may be interested to know that there were no significant differences between the 49 HR and 49 LR infants on either of the key findings. We will supply the data to illustrate these similarities if requested, A and B, a fact illustrated below for the benefit of the prior reviewers even though we have removed from the proposed paper all the data on infants at risk.**

**The similarity of the risk groups on CBR may be surprising because various measures of canonical babbling seem to be good predictors of later ASD symptomatology (Paul et al., 2011), are very important in certain other clinical cases (deafness in particular Eilers & Oller, 1994; Nathani et al., 2007; Oller & Eilers, 1988), and there is even evidence of possible babbling differences between infants later diagnosed with ASD and infants with no diagnosis (Patten et al., 2014). Still, there are lots of other factors (beyond babbling) that are also involved in the acquisition of speech and language. It remains to be seen whether the canonical babbling measures we have used actually play a particularly important role in later language development in ASD. We will be investigating such issues in subsequent papers analyzing the data as they continue to come in. We resolved long ago to withhold publication of data comparing outcomes for the infants who later receive or do not receive a diagnosis of ASD until we have a substantial number of infants who have been conclusively diagnosed. We will have >300 infants in the final dataset, with about 20% receiving ASD diagnoses.**

**Our conclusion: The empirical focus of our paper should be the robustly different distributions of TT and VP and the relation between increases in CBR corresponding to both factors, TT and VP. The new empirical focus is accompanied by a rationale based on the importance of these findings in enhancing perspectives on the reasons protophones/babbling exist—they appear to function both as copious fitness signals to caregivers and as a foundation for development of the speech capacity.**

Both reviewers raise the issue of whether the ‘high-risk for autism group’ can be considered as low in social motivation so this should be addressed from the outset as it impacts on the formulation of your research questions.

**We agree that lower social motivation in HR infants is uncertain. We have resolved to leave out the HR group and provide a more comprehensive treatment of the risk groups along with ASD diagnosis of a subset of the subjects in a separate paper when data collection is complete.**

I also agree with Reviewer 2 that the information about model structure and results should be included into the main text as this is what is being interpreted in the Discussion. In my own view, this is especially important since readers need to understand the motivation behind your choice of GEEs as opposed to LMEMs, whether and how this affects your method of contrast coding and its implications for whether effects are interpreted as simple vs. main effects.

Furthermore, you mention several different models but do not seem to provide model comparison data. From your analysis code on the OSF I can see that some models coded age numerically and some used treatment coding of age groups, but it is not clear which models were selected for reporting and why. Overall, the section called ‘Statistical Approach’ needs to be significantly expanded to include revised parts of what is now Supplementary Information and more clarity with respect to your analysis strategy. This seems especially important as some models did show an effect of TT and some did not.

**The statistical analysis section has been clarified and adjusted to the revised aims, while the analysis approach has been simplified by eliminating the risk factor.**

This may be related to the lack of clarity about your segment coding for Turn Taking vs Vocal Play and its treatment in the statistical models. I could grasp the distribution of TT vs VP across sessions only after consulting the cross-tabulation in the Supplementary Materials so to avoid confusion by the readers I strongly suggest moving the first two tables from Appendix A into the main text and remove Table 3 which strikes me as unhelpful as it obscures the relationship between TT and VP. It may make sense to streamline the cross-tabulation based on your dichotomization of the TT and VP ratings resulting in four types of segments: 1) lowTT/lowVP, 2) lowTT/highVP, 3) highTT/lowVP, and 4) highTT/highVP. Even though the highTT/highVP combination does not occur the lowTT/lowVP combination does and so I doubt that it is justified to analyse the data separately for TT and VP rather than run a model that includes both fixed effects. Your r-code on the OSF suggests that you did run such a model so please motivate which models you present in the paper and why or present a multiverse analysis.

**We see the point. We have streamlined the information in keeping with the decision to make the TT/VP distributional difference a main focus of the paper. A new table has been provided and details are provided by age of infants in the Supplementary Material. The refocused and revised paper now portrays the distributional differences of TT and VP as a primary finding in the Results section, with the data portrayed in figures and a table.**

Both reviewers make further  helpful comments on how to interpret findings with respect to the HR and LR groups for autism which I urge you to take into consideration. Unfortunately, a 3^rd^ reviewer, who is an expert in early infant vocalizations rather than in autism, was unable to provide a review. Still, I feel the paper needs a clearer theoretical interpretation for what your data show with respect to the role of social vs endogenous motivations for canonical babbling and how these two drivers may interact. You state in line 390 that the effect was larger for VP but you don’t seem to provide statistical evidence to back this up.

Given the multiple statistical models you have run it is not clear what would constitute such evidence so I urge to provide a clearer link between you statistical modelling and your theoretical conclusions with respect to the motivations that may drive canonical babbling. In light of the reviewers’ comments on whether the HR group can be assumed to have lower social motivation or not you may want to de-emphasise the interpretation of the null effects of risk group in favour of a clearer theoretical discussion of the two types of babbling motivation.

**Agreed. We have restructured to supply a clearer portrayal of our presumed “motivations” for babbling. Of course what the paper can present unambiguously is observations showing a dramatic difference between the rates of TT and VP. The interpretation of those differences may become a matter of debate, but our approach invokes infrastructural thinking about how vocal repertoires develop. The traditional assumption is that infants primarily learn to produce speech and speech-like sounds by imitation of caregiver utterances. This idea is hard to support in the context of data showing that infants actually produce immediate vocal imitations extremely rarely and overwhelmingly produce pre-speech vocalizations when they are not engaged in interaction at all, but instead during periods of exploratory vocal play. The revision makes clear, however, that we view vocal interaction as critical even if it is rare. Further we argue that during vocal interaction, it is not justified to conclude infants are not engaged in a kind of active (endogenously driven) exploration, in this case an exploration of how their vocal repertoire can be utilized (and perhaps expanded) in periods when they are also attending to and responding to vocalizations of their interlocutors.**

Both reviewers have made many other helpful comments that I hope you will consider in your revision.

One minor point: For the first two models reported in the Appendix, you base your predictions on the social motivation theory which seems to be wrong with respect to the model containing VP as a predictor as this clearly pertains to the endogenous/play theory (I suspect this is a copying error in line 227 of the SI).

**Thanks for the heads up.**

Please submit your revised manuscript by May 12 2022 11:59PM. If you will need more time than this to complete your revisions, please reply to this message or contact the journal office at [plosone@plos.org](mailto:plosone@plos.org). When you're ready to submit your revision, log on to [https://www.editorialmanager.com/pone/](https://nam02.safelinks.protection.outlook.com/?url=https%3A%2F%2Fwww.editorialmanager.com%2Fpone%2F&data=04%7C01%7Ckoller%40memphis.edu%7Cbd95655a72514d51170f08da1195bee3%7Cae145aeacdb2446ab05a7858dde5ddba%7C0%7C0%7C637841633168166047%7CUnknown%7CTWFpbGZsb3d8eyJWIjoiMC4wLjAwMDAiLCJQIjoiV2luMzIiLCJBTiI6Ik1haWwiLCJXVCI6Mn0%3D%7C2000&sdata=v0Ei5qwz2HrJ1iqcMc0TrvykwkxX4rUgIELljS5xsEk%3D&reserved=0) and select the 'Submissions Needing Revision' folder to locate your manuscript file.

Please include the following items when submitting your revised manuscript:

- A rebuttal letter that responds to each point raised by the academic editor and reviewer(s). You should upload this letter as a separate file labeled 'Response to Reviewers'.
- A marked-up copy of your manuscript that highlights changes made to the original version. You should upload this as a separate file labeled 'Revised Manuscript with Track Changes'.
- An unmarked version of your revised paper without tracked changes. You should upload this as a separate file labeled 'Manuscript'.

If you would like to make changes to your financial disclosure, please include your updated statement in your cover letter. Guidelines for resubmitting your figure files are available below the reviewer comments at the end of this letter.

If applicable, we recommend that you deposit your laboratory protocols in protocols.io to enhance the reproducibility of your results. Protocols.io assigns your protocol its own identifier (DOI) so that it can be cited independently in the future. For instructions see: [https://journals.plos.org/plosone/s/submission-guidelines#loc-laboratory-protocols](https://nam02.safelinks.protection.outlook.com/?url=https%3A%2F%2Fjournals.plos.org%2Fplosone%2Fs%2Fsubmission-guidelines%23loc-laboratory-protocols&data=04%7C01%7Ckoller%40memphis.edu%7Cbd95655a72514d51170f08da1195bee3%7Cae145aeacdb2446ab05a7858dde5ddba%7C0%7C0%7C637841633168166047%7CUnknown%7CTWFpbGZsb3d8eyJWIjoiMC4wLjAwMDAiLCJQIjoiV2luMzIiLCJBTiI6Ik1haWwiLCJXVCI6Mn0%3D%7C2000&sdata=de3K1VhxW2ArQYbbqQ%2FlbY0O%2BrvJuvN3c7wukU0MA80%3D&reserved=0). Additionally, PLOS ONE offers an option for publishing peer-reviewed Lab Protocol articles, which describe protocols hosted on protocols.io. Read more information on sharing protocols at [https://plos.org/protocols?utm_medium=editorial-email&utm_source=authorletters&utm_campaign=protocols](https://nam02.safelinks.protection.outlook.com/?url=https%3A%2F%2Fplos.org%2Fprotocols%3Futm_medium%3Deditorial-email%26utm_source%3Dauthorletters%26utm_campaign%3Dprotocols&data=04%7C01%7Ckoller%40memphis.edu%7Cbd95655a72514d51170f08da1195bee3%7Cae145aeacdb2446ab05a7858dde5ddba%7C0%7C0%7C637841633168166047%7CUnknown%7CTWFpbGZsb3d8eyJWIjoiMC4wLjAwMDAiLCJQIjoiV2luMzIiLCJBTiI6Ik1haWwiLCJXVCI6Mn0%3D%7C2000&sdata=AElS4w9DlgbY8dKlrg2hy2xS3UWNwFISgyFyyf9Um%2BA%3D&reserved=0).

We look forward to receiving your revised manuscript.

Kind regards,

Vera Kempe
Academic Editor
PLOS ONE

Journal Requirements:

When submitting your revision, we need you to address these additional requirements.

1. Please ensure that your manuscript meets PLOS ONE's style requirements, including those for file naming. The PLOS ONE style templates can be found at 
[https://journals.plos.org/plosone/s/file?id=wjVg/PLOSOne_formatting_sample_main_body.pdf](https://nam02.safelinks.protection.outlook.com/?url=https%3A%2F%2Fjournals.plos.org%2Fplosone%2Fs%2Ffile%3Fid%3DwjVg%2FPLOSOne_formatting_sample_main_body.pdf&data=04%7C01%7Ckoller%40memphis.edu%7Cbd95655a72514d51170f08da1195bee3%7Cae145aeacdb2446ab05a7858dde5ddba%7C0%7C0%7C637841633168166047%7CUnknown%7CTWFpbGZsb3d8eyJWIjoiMC4wLjAwMDAiLCJQIjoiV2luMzIiLCJBTiI6Ik1haWwiLCJXVCI6Mn0%3D%7C2000&sdata=E6ZvqYEIm%2FqAUjjvX1lIb8uTBTefNl07GWiDoALNrWw%3D&reserved=0) and [https://journals.plos.org/plosone/s/file?id=ba62/PLOSOne_formatting_sample_title_authors_affiliations.pdf](https://nam02.safelinks.protection.outlook.com/?url=https%3A%2F%2Fjournals.plos.org%2Fplosone%2Fs%2Ffile%3Fid%3Dba62%2FPLOSOne_formatting_sample_title_authors_affiliations.pdf&data=04%7C01%7Ckoller%40memphis.edu%7Cbd95655a72514d51170f08da1195bee3%7Cae145aeacdb2446ab05a7858dde5ddba%7C0%7C0%7C637841633168166047%7CUnknown%7CTWFpbGZsb3d8eyJWIjoiMC4wLjAwMDAiLCJQIjoiV2luMzIiLCJBTiI6Ik1haWwiLCJXVCI6Mn0%3D%7C2000&sdata=PkJoulYuyrzjOfA9Uuttq1vDibzU9HlboJQttR8Zg9o%3D&reserved=0)

**Edited.**

2. In your Data Availability statement, you have not specified where the minimal data set underlying the results described in your manuscript can be found. PLOS defines a study's minimal data set as the underlying data used to reach the conclusions drawn in the manuscript and any additional data required to replicate the reported study findings in their entirety. All PLOS journals require that the minimal data set be made fully available. For more information about our data policy, please see [http://journals.plos.org/plosone/s/data-availability](https://nam02.safelinks.protection.outlook.com/?url=http%3A%2F%2Fjournals.plos.org%2Fplosone%2Fs%2Fdata-availability&data=04%7C01%7Ckoller%40memphis.edu%7Cbd95655a72514d51170f08da1195bee3%7Cae145aeacdb2446ab05a7858dde5ddba%7C0%7C0%7C637841633168166047%7CUnknown%7CTWFpbGZsb3d8eyJWIjoiMC4wLjAwMDAiLCJQIjoiV2luMzIiLCJBTiI6Ik1haWwiLCJXVCI6Mn0%3D%7C2000&sdata=l0E9HsRCgJss2PVhmFbwI7ocNhDpYKU1SHDRndkfqzE%3D&reserved=0).

Upon re-submitting your revised manuscript, please upload your study’s minimal underlying data set as either Supporting Information files or to a stable, public repository and include the relevant URLs, DOIs, or accession numbers within your revised cover letter. For a list of acceptable repositories, please see [http://journals.plos.org/plosone/s/data-availability#loc-recommended-repositories](https://nam02.safelinks.protection.outlook.com/?url=http%3A%2F%2Fjournals.plos.org%2Fplosone%2Fs%2Fdata-availability%23loc-recommended-repositories&data=04%7C01%7Ckoller%40memphis.edu%7Cbd95655a72514d51170f08da1195bee3%7Cae145aeacdb2446ab05a7858dde5ddba%7C0%7C0%7C637841633168166047%7CUnknown%7CTWFpbGZsb3d8eyJWIjoiMC4wLjAwMDAiLCJQIjoiV2luMzIiLCJBTiI6Ik1haWwiLCJXVCI6Mn0%3D%7C2000&sdata=cr0ZC9h%2FxGTj791Jfwft3KaEogwpQvMnuE0Ubh%2FDTvE%3D&reserved=0). Any potentially identifying patient information must be fully anonymized.

Important: If there are ethical or legal restrictions to sharing your data publicly, please explain these restrictions in detail. Please see our guidelines for more information on what we consider unacceptable restrictions to publicly sharing data: [http://journals.plos.org/plosone/s/data-availability#loc-unacceptable-data-access-restrictions](https://nam02.safelinks.protection.outlook.com/?url=http%3A%2F%2Fjournals.plos.org%2Fplosone%2Fs%2Fdata-availability%23loc-unacceptable-data-access-restrictions&data=04%7C01%7Ckoller%40memphis.edu%7Cbd95655a72514d51170f08da1195bee3%7Cae145aeacdb2446ab05a7858dde5ddba%7C0%7C0%7C637841633168166047%7CUnknown%7CTWFpbGZsb3d8eyJWIjoiMC4wLjAwMDAiLCJQIjoiV2luMzIiLCJBTiI6Ik1haWwiLCJXVCI6Mn0%3D%7C2000&sdata=zZXJ1I7MzHv5m0SZ%2Fea8BwpNlUeFq%2B%2ByjoNCVMcZXE4%3D&reserved=0). Note that it is not acceptable for the authors to be the sole named individuals responsible for ensuring data access.

We will update your Data Availability statement to reflect the information you provide in your cover letter.

**The dataset analyzed in the present study will be made available on the Open Science Framework at the point of acceptance at https://osf.io/ery6b/. We do not have the requisite permissions for the audio recordings to be shared publicly (subjects are not entirely unidentifiable in the recordings), so recordings cannot be made available for sharing.**

3. Please upload a copy of Figure 2, to which you refer in your text on page 14. If the figure is no longer to be included as part of the submission, please remove all reference to it within the text.

**All figures revised and uploaded.**

4. Please include captions for your Supporting Information files at the end of your manuscript, and update any in-text citations to match accordingly. Please see our Supporting Information guidelines for more information: [http://journals.plos.org/plosone/s/supporting-information](https://nam02.safelinks.protection.outlook.com/?url=http%3A%2F%2Fjournals.plos.org%2Fplosone%2Fs%2Fsupporting-information&data=04%7C01%7Ckoller%40memphis.edu%7Cbd95655a72514d51170f08da1195bee3%7Cae145aeacdb2446ab05a7858dde5ddba%7C0%7C0%7C637841633168166047%7CUnknown%7CTWFpbGZsb3d8eyJWIjoiMC4wLjAwMDAiLCJQIjoiV2luMzIiLCJBTiI6Ik1haWwiLCJXVCI6Mn0%3D%7C2000&sdata=baLCWNezk%2FOsQaylcY9aJV8gPrRRFN31iiEDKx3Exq8%3D&reserved=0).
**Added.**

[Note: HTML markup is below. Please do not edit.]

Reviewers' comments:

Reviewer's Responses to Questions

Comments to the Author

1. Is the manuscript technically sound, and do the data support the conclusions?

The manuscript must describe a technically sound piece of scientific research with data that supports the conclusions. Experiments must have been conducted rigorously, with appropriate controls, replication, and sample sizes. The conclusions must be drawn appropriately based on the data presented.

Reviewer #1: Partly

Reviewer #2: Partly

2. Has the statistical analysis been performed appropriately and rigorously?

Reviewer #1: Yes

Reviewer #2: Yes

3. Have the authors made all data underlying the findings in their manuscript fully available?

The [PLOS Data policy](https://nam02.safelinks.protection.outlook.com/?url=http%3A%2F%2Fwww.plosone.org%2Fstatic%2Fpolicies.action%23sharing&data=04%7C01%7Ckoller%40memphis.edu%7Cbd95655a72514d51170f08da1195bee3%7Cae145aeacdb2446ab05a7858dde5ddba%7C0%7C0%7C637841633168166047%7CUnknown%7CTWFpbGZsb3d8eyJWIjoiMC4wLjAwMDAiLCJQIjoiV2luMzIiLCJBTiI6Ik1haWwiLCJXVCI6Mn0%3D%7C2000&sdata=w%2BwdSAlqfha0iClUf2jB1hJsKLVFVnjFWFh2iJP5vJg%3D&reserved=0) requires authors to make all data underlying the findings described in their manuscript fully available without restriction, with rare exception (please refer to the Data Availability Statement in the manuscript PDF file). The data should be provided as part of the manuscript or its supporting information, or deposited to a public repository. For example, in addition to summary statistics, the data points behind means, medians and variance measures should be available. If there are restrictions on publicly sharing data—e.g. participant privacy or use of data from a third party—those must be specified.

Reviewer #1: Yes

Reviewer #2: Yes

4. Is the manuscript presented in an intelligible fashion and written in standard English?

PLOS ONE does not copyedit accepted manuscripts, so the language in submitted articles must be clear, correct, and unambiguous. Any typographical or grammatical errors should be corrected at revision, so please note any specific errors here.

Reviewer #1: Yes

Reviewer #2: Yes

5. Review Comments to the Author

Please use the space provided to explain your answers to the questions above. You may also include additional comments for the author, including concerns about dual publication, research ethics, or publication ethics. (Please upload your review as an attachment if it exceeds 20,000 characters)

Reviewer #1: The article is very interesting and well written. It compares canonical babbling in infants at high or low risk for ASD in different socio-communicative situations. The main issue is that the conclusions driven from the results are limited to high vs. low risk, but the authors did not discuss that a certain - most likely high - proportion of infants at high risk for ASD will not be diagnosed with ASD later in their lives. This leads me to the following question: Why should high risk children who develop typically differ from low risk children in canonical babbling? Do you know how many of the high risk infants were later diagnosed with ASD? I do understand if the outcome of the children is not known to date, but it should be discussed as a limitation that it is unkown how many of the high risk children actually have ASD and how many of the low risk children actually develop typically. Please especially consider to rewrite the sentence on page 18, lines 379-381, and the first paragrah on page 20, taking into account the limitation mentioned above. Did you consider any inclusion criteria concerning pregnancy/delivery, i.e., preterm/term birth, potential complications, birth weight and size, head circumference, APGAR score, etc.? Are there group differences concerning these aspects?

**Because of our major restructuring to focus only on the LR infant data, these issues are no longer germane. We will answer these important questions on HR children in a follow-up paper.**

Minor issues:
Page 3, line 54: Would it be more accurate to use wellbeing instead of wellness?

**Although the section this comment was referring to has now been** **substantially restructured, the terms wellness and well-being both appear in the manuscript as alternatives to fitness. This is a stylistic decision—writing fitness every time it might be appropriate just seemed too repetitive.**

Page 10, line 196: Please rather use "from the first study month". Without the word "study" one may misunderstand it as the first month of life.

**This section is now substantially revised and this phrase is removed.**

Page 11, lines 212-214: Please clarify the procedure of selecting 8 out of 21 segments. Did you take care for age-matching?

**We have substantially restructured the section describing this procedure into what is now under the heading Segment Selection and Coders in Materials and Methods.**

Page 13, lines 271-278: Please clarify: How can a segment contain more than half the time VP AND TT? In this case, VP and TT would need to occur simultaneously. Is this possible?

**Good question, and we see that it’s important to clarify. We have added text to this point in the Coding Categories section of Materials and Methods.**

Reviewer #2: Thank you for the opportunity to review this manuscript, which presents interesting canonical babbling data and interprets it through an evolutionary developmental biology lens. This is a good study with strong methods and interesting results, but in the current format, some of the major claims in the discussion are unsupported by data presented in the manuscript. Specifically, significant space is dedicated in the discussion to interpreting the implications of the findings on the role of social motivation in babbling development, which is substantiated by null interactions that are presented only in the supplementary material. I think this can be largely resolved by moving these analyses to the main text. Based on the discussion, interactions with risk appear to be a main focus of the paper, and I think the paper would be strengthened if the introduction, methods, and results were edited to reflect that. Major and minor comments follow.

**We agree. Because of the substantial revisions to the paper including the participants included, we have de-emphasized the interpretation of the social motivation theory.**

Major Comments:

Abstract
The fact that the sample includes infants at high and low risk for autism should be mentioned in the abstract.

**The revised paper now only includes LR infants.**

Introduction
Why is it taken as a given that babbling is a signal of fitness rather than simply selected for because it is a good way develop language, which is itself beneficial?

**We hope the substantially revised introduction and discussion offer additional perspective on this point. The key issue is that in modern times babbling does appear (as the reviewer suggests) to supply a necessary foundation for language, and its maintenance may indeed (as the reviewer suggests) be supported by that fact. However, evolutionary theory makes clear that any evolving capacity must be selected in accord with pressures that apply at the time of the appearance of the new capacity. Babbling could not have been evolved by a selection pressure for language at the beginning of the evolutionary process that would ultimately yield language because language did not yet exist. Babbling had to have some advantage of its own at that point. Once established, however, babbling could have formed a foundation, in accord with evo-devo theory, for additional capacities that led to language, and indeed the naturally selecting pressures could have become multiple. We argue that the selection pressure on fitness signaling remains operative even though (as the reviewer suggests) the need for language in modern humans presumably also contributes nowadays to the maintenance of babbling in humans.**

If an entire section of the discussion will be dedicated to the lack of risk*TT interaction, this should be listed as a hypothesis. The risk*VP interaction hypothesis should probably be listed, as well.

**The paper no longer addresses risk.**

Methods:
Regarding the low-high median split of SES: what is the point of this? It doesn’t seem like these groups are actually used for anything in the analysis, and as they are, they do not communicate much about the SES levels of the groups. It would be more helpful to anchor where that split fell in terms of the report of maternal education, or perhaps just report (maybe in the supplement) the maternal education levels reported by group.

**We have added an additional discussion on both sex and SES in Supplementary Information section A.**

I’m confused about the segment selection. First: “From these 21, eight segments, all having been coded as showing high volubility (infant protophone rate) and a range from high to low infant-directed speech were selected from each recording, yielding a total of 3799 segments.” I think this sentence is missing a word. Is this saying that from the 21 randomly chosen segments, the eight segments with the highest volubility for each participant was selected? If it was not based on the highest volubility, it would be good to clarify how the 8 segments were picked.

**We have added further clarification on the selection of segments to the now-labeled section “Segment Selection and Coders” in Materials and Methods.**

Second, I did not understand until I read the results that if an infant had multiple days of recordings that fell within a single “timepoint,” all of the recordings were used, meaning that infants had varying numbers of segments within a timepoint, and some infants contributed data ~1 month apart to the same timepoint. It would help the reader to make this more explicit in the methods.

**We have added a statement clarifying this information to the Audio Recordings section of Materials and methods.**

Regarding the statistical approach, I don’t understand why the information about the GEEs was relegated to the supplement, but it should absolutely be in the main text. It was challenging to figure out what the analyses actually consisted of from the main text. Moving those two paragraphs to the main text would be good.

**We hope that the substantially revised manuscript (and Results) have streamlined these findings.**

Results

Include the full interaction results, since these are heavily interpreted in the discussion.

**With the substantial revisions we no longer have interaction data on risk to report. Interactions with Age are treated in Results and Discussion.**

Discussion
Social motivation section: It seems like a misreading of the social motivation hypothesis of autism to assume that the high risk group as a whole should be considered to have reduced social motivation. While it is reasonable to hypothesize that reduced social motivation is an endophenotype of autism, with an intermediate phenotype observed in those high risk siblings who do not ultimately receive an ASD diagnosis, it could alternatively be predicted that higher levels of social motivation among HR-no-autism-outcome siblings is precisely what protects them from an eventual ASD diagnosis. Thus, the HR group overall is not necessarily expected to have lower social motivation, even if the HR-ASD-outcome infants do.

**The social motivation theory no longer has the role it had in the prior version of the paper because we have eliminated the risk factor. We hope to address these points in a future paper.**

Line 394 “The findings for CBRs during TT (alongside no major differences between risk groups) suggest similar levels of social motivation in both groups, with all infants showing the tendency to produce higher CBRs during segments rated as having any TT compared to those rated as TT.” These findings are not presented in the main text. Include the interaction effects to make this claim.

**With the substantial revisions we no longer have interaction data to report about risk.**

Also, there is no discussion of the fact that the authors examined the HR group as a whole, where there may be a more clear difference if they separately examined the HR infants who do and do not go on to receive an ASD diagnosis. For example, line 417: “Our results suggest that the most advanced prelinguistic vocal forms (i.e., canonical syllables) produced during early face-to-face interactions may be robust even in the face of a potentially elevated likelihood for social communication deficits.” I don’t really understand what this is saying. The HR group isn’t potentially elevated in risk, they are elevated in risk. But within that group are clear subgroups (that the authors may have access to) of infants who do or do not go on to actually have social communication deficits.

**We hope to address these points in a future paper where a much larger quantity of data will be used to address risk of ASD.**

This paragraph/sentence can’t stand on its own (line 458) Interestingly, a preliminary analysis on VP data revealed a significant difference in CBRs between Risk groups over time, but only during periods of high VP (p = .039, b = .06, see SI Appendix C for additional analyses). Either mention the results of these preliminary analyses in the results and expand on this further, or don’t. As it stands, this sentence doesn’t even give the direction of effects.

**We have removed this statement altogether with the removal of the HR group data.**

Conclusion:
“Our results on the risk groups offer support for a potentially robust social motivation in infancy to produce higher rates of canonical syllables during interaction, even in the presence of an elevated likelihood for social communication deficits.” Again, this sentence is not substantiated by results presented in the paper as it stands, but would be fine if the null interaction is included.

**We have removed this statement altogether with the removal of the HR group data.**

Minor Comments
Intro:
“And it is especially important to determine the role of canonical babbling, because it is so much like mature speech, during these different circumstances.” This sentence could be strengthened to more clearly articulate the aim of the study.

**We have substantially revised this section, including the aims of the paper.**

This sentence was really hard to parse (intro line 118) “Our assumption is that both social and exploratory motivations are involved in the production of protophones—even during social interaction, one can imagine that playful exploration of sounds and perhaps relations of the protophones with the sounds of the interacting other speakers may motivate infant activity.”

**We have substantially revised this section and hope the revisions have provided clarity on this point.**

Methods:
Missing a word (line 256) “For example, a TT rating of 5 was applied to segments where a caregiver was clearly speaking to the infant, and the infant was protophones in an apparent back and forth vocal interaction for essentially the whole segment.”

**The new methods supply a great deal more detail on the how TT and VP were rated.**

Results:
Line 324 “indicating an increased production of canonical babbling during periods with high social vocal activity compared to low social vocal activity (p < .001, b = .04).” This should say “any social vocal activity compared to no social vocal activity” to accurately reflect the split of data that was examined.

**Thank you for pointing this out, we have substantially revised this section and hopefully clarified this distinction throughout.**

Discussion:
Line 380 “may be more resistant to cognitive effects of risk than has been expected.” I don’t understand what is meant by “cognitive effects of risk”

**Sentence removed.**

6. PLOS authors have the option to publish the peer review history of their article ([what does this mean?](https://nam02.safelinks.protection.outlook.com/?url=https%3A%2F%2Fjournals.plos.org%2Fplosone%2Fs%2Feditorial-and-peer-review-process%23loc-peer-review-history&data=04%7C01%7Ckoller%40memphis.edu%7Cbd95655a72514d51170f08da1195bee3%7Cae145aeacdb2446ab05a7858dde5ddba%7C0%7C0%7C637841633168166047%7CUnknown%7CTWFpbGZsb3d8eyJWIjoiMC4wLjAwMDAiLCJQIjoiV2luMzIiLCJBTiI6Ik1haWwiLCJXVCI6Mn0%3D%7C2000&sdata=BnhQhEiZqo8FiqOiKO%2BtX%2FRqC9TvoIQ0mSaynUJGH3w%3D&reserved=0)). If published, this will include your full peer review and any attached files.


If you choose “no”, your identity will remain anonymous but your review may still be made public.


Do you want your identity to be public for this peer review? For information about this choice, including consent withdrawal, please see our [Privacy Policy](https://nam02.safelinks.protection.outlook.com/?url=https%3A%2F%2Fwww.plos.org%2Fprivacy-policy&data=04%7C01%7Ckoller%40memphis.edu%7Cbd95655a72514d51170f08da1195bee3%7Cae145aeacdb2446ab05a7858dde5ddba%7C0%7C0%7C637841633168166047%7CUnknown%7CTWFpbGZsb3d8eyJWIjoiMC4wLjAwMDAiLCJQIjoiV2luMzIiLCJBTiI6Ik1haWwiLCJXVCI6Mn0%3D%7C2000&sdata=qZt%2FkhTFCl0YgZ3dbwBGKGrR7I6Wj7My%2B1WQTvhcThw%3D&reserved=0).

Reviewer #1: No

Reviewer #2: No

[NOTE: If reviewer comments were submitted as an attachment file, they will be attached to this email and accessible via the submission site. Please log into your account, locate the manuscript record, and check for the action link "View Attachments". If this link does not appear, there are no attachment files.]

While revising your submission, please upload your figure files to the Preflight Analysis and Conversion Engine (PACE) digital diagnostic tool, [https://pacev2.apexcovantage.com/](https://nam02.safelinks.protection.outlook.com/?url=https%3A%2F%2Fpacev2.apexcovantage.com%2F&data=04%7C01%7Ckoller%40memphis.edu%7Cbd95655a72514d51170f08da1195bee3%7Cae145aeacdb2446ab05a7858dde5ddba%7C0%7C0%7C637841633168166047%7CUnknown%7CTWFpbGZsb3d8eyJWIjoiMC4wLjAwMDAiLCJQIjoiV2luMzIiLCJBTiI6Ik1haWwiLCJXVCI6Mn0%3D%7C2000&sdata=8ygDC4v3I42hiMbOowjvJR7iAVkTQMWu%2FZsSErNf6G0%3D&reserved=0). PACE helps ensure that figures meet PLOS requirements. To use PACE, you must first register as a user. Registration is free. Then, login and navigate to the UPLOAD tab, where you will find detailed instructions on how to use the tool. If you encounter any issues or have any questions when using PACE, please email PLOS at [figures@plos.org](mailto:figures@plos.org). Please note that Supporting Information files do not need this step.

**References**

Eilers, R. E., & Oller, D. K. (1994). Infant vocalizations and the early diagnosis of severe hearing impairment. *Journal of Pediatrics*, *124*, 199–203.

Locke, J. L. (2006). Parental selection of vocal behavior: Crying, cooing, babbling, and the evolution of language. *Human Nature*, *17*(2), 155–168. doi: 10.1007/s12110-006-1015-x.

Nathani, S., Oller, D. K., & Neal, A. R. (2007). On the robustness of vocal development: An examination of infants with moderate-to-severe hearing loss and additional risk factors. *Journal of Speech Language and Hearing Research*, *50*(6), 1425. doi: 10.1044/1092-4388(2007/099).

Oller, D. K., Caskey, M., Yoo, H., Bene, E. R., Jhang, Y., Lee, C.-C., Bowman, D. D., Long, H. L., Buder, E. H., & Vohr, B. (2019). Preterm and full-term infant vocalization and the origin of language. *Scientific Reports*, *9*(1), 14734. doi: 10/gnzf8m.

Oller, D. K., & Eilers, R. E. (1988). The role of audition in infant babbling. *Child Development*, *59*(2), 441–449. doi: 10.1111/j.1467-8624.1988.tb01479.x.

Oller, D. K., & Griebel, U. (2005). Contextual freedom in human infant vocalization and the evolution of language. In *Evolutionary perspectives on human development* (Vol. 2). SAGE Publications.

Oller, D. K., Griebel, U., Iyer, S. N., Jhang, Y., Warlaumont, A. S., Dale, R., & Call, J. (2019). Language origins viewed in spontaneous and interactive vocal rates of human and bonobo infants. *Frontiers in Psychology*, *10*, 729. doi: 10.3389/fpsyg.2019.00729.

Oller, D. K., Griebel, U., & Warlaumont, A. S. (2016). Vocal development as a guide to modeling the evolution of language. *Topics in Cognitive Science*, *8*(2), 382–392.

Oller, D. K., Ramsay, G., Bene, E., Long, H. L., & Griebel, U. (2021). Protophones, the precursors to speech, dominate the human infant vocal landscape. *Philosophical Transactions of the Royal Society B: Biological Sciences*, *376*(1836), 20200255. doi: 10.1098/rstb.2020.0255.

Patten, E., Belardi, K., Baranek, G. T., Watson, L. R., Labban, J. D., & Oller, D. K. (2014). Vocal patterns in infants with autism spectrum disorder: Canonical babbling status and vocalization frequency. *Journal of Autism and Developmental Disorders*, 1–16. doi: 10.1007/s10803-014-2047-4.

Paul, R., Fuerst, Y., Ramsay, G., Chawarska, K., & Klin, A. (2011). Out of the mouths of babes: Vocal production in infant siblings of children with ASD. *Journal of Child Psychology and Psychiatry and Allied Disciplines*, *52*(5), 588–598. doi: 10.1111/j.1469-7610.2010.02332.x.

Warlaumont, A. S., Richards, J. A., Gilkerson, J., & Oller, D. K. (2014). A social feedback loop for speech development and its reduction in autism. *Psychological Science*, *25*, 1314–1324. doi: 10.1177/0956797614531023.
