# Supplementary material for: Perspectives on the origin of language: Infants vocalize most during independent vocal play but produce their most speech-like vocalizations during turn taking
Source: PLoS One. 2022 Dec 30;17(12):e0279395. doi: 10.1371/journal.pone.0279395 (PMC9803194; doi:10.1371/journal.pone.0279395)
Supplement: Attachment — Submitted filename: Response to Reviewers.docx [file pone.0279395.s009.docx]

Response letter for PONE-D-22-03801R1

Infants vocalize most during independent vocal play but produce their most speech-like vocalizations during vocal turn taking

Dear Editor (Professor Kempe):

**Our responses to reviewer comments are in boldface throughout the following letter.**

Reviewer #2: Thank you for the opportunity to review this substantially revised manuscript. By shifting the focus away from evaluating the social motivation hypothesis and eliminating the infants with elevated likelihood for autism, the authors have addressed many of my initial concerns (and I look forward to their future paper examining these relationships in autism). The new manuscript is much more streamlined and provides significantly clearer rationale for the tests conducted and conclusions drawn, with a goal of evaluating the role of endogenous vocal activity in vocal learning. My main concerns at this stage are 1) that the selection of high volubility segments may influence the main conclusions drawn, and 2) that the low coder agreement on the Vocal Play (VP) and Turn Taking (TT) measures may significantly impact conclusions drawn about canonical babbling ratios (CBR) during these periods in ways that are not acknowledged. These concerns are explained in more detail below, along with some more minor suggestions.

Abstract
It would really help to spell out that VP and TT are neither mutually exclusive in segments nor encompass all kinds of vocalizations. At the least, changing “Periods of vocal play and turn-taking corresponded to elevated levels of the most advanced protophones (canonical babbling),” to “Periods of vocal play and turn-taking corresponded to elevated levels of the most advanced protophones (canonical babbling) relative to periods without vocal play or without turn-taking,” would help.

**Thanks for reviewing of our revision and for the probing commentary.**

**We have inserted the reviewer’s suggested sentence in the abstract. In accord with how we understand the more general concerns about the abstract, we think they are best addressed in revisions to the section on Testable Propositions, where, in accord with the reviewer’s suggestion, we have added a paragraph specifying 1) that VP and TT are not mutually exclusive in 5 min segments; that 2) the term protophones is used in the paper to encompass only the very frequently occurring phonated sounds: vocants, squeals and growls, wherein both canonical and non-canonical syllables can occur; that 3) our protophone counts in this paper do not include ingressive sounds or a variety of non-phonated sounds (e.g., whispers, clicks, voiceless friction sounds, etc.), which occur far less often than the sounds included in our protophone counts; and that 4) some protophones are not treated as potential VP; in particular whining (estimated by the complaints item in the Phase 2 questionnaire) is excluded from VP because whining can be interpreted as being motivated more by distress than by any exploratory inclination.**

Testable Propositions
Why use “proposition” instead of “hypothesis?”

**The choice is based on the fact that this is not experimental, but longitudinal observational research, where we think it makes sense to emphasize that strict hypothesis testing is not involved, and where control over a variety of potential extraneous variables is not possible because there is no possibility of random assignment to the groups to be compared. In such observational research we regularly present analyses that offer varying perspectives on outcomes rather than relying on single hypothesis evaluation, as is typically done in experimental research.**

Proposition 2 – At this point, I did not understand how can a segment could have no VP without simply being TT. This is explained in great length later in the methods section, but should be briefly mentioned here (e.g., a brief mention of things like complaints).

**Clarified under new verbiage in the new paragraph in Testable Propositions.**

Participants
I still feel that some direct information about SES should be provided – what measure of SES was used to create the median split, and what was the median and range of the cohort reported in this paper?

**Mother’s educational level was the proxy for SES, as stated in the prior version of the ms. The measure consisted of reported number of years of education ranging from 13 to 25 with a median of 18. This information has been inserted in the revised text.**

Segment selection
Why were segments selected for high infant volubility? This seems like a potentially crucial decision that could very much affect the main outcomes of interest. I would hypothesize that segments selected for high infant volubility are more likely to be perceived as including VP. This affects proposition 1 (relative rates of VP versus TT).

**As specified in Methods, we selected 21 segments randomly in Phase 1. Only in Phase 2 did we obtain counts of canonical and non-canonical syllables. Consequently our study of CBR *could only focus on Phase 2 segments*, which had been limited in number due to coding costs projected at the beginning of the grant that funded the effort—we did not have the funds to code all 21 segments in Phase 2. Also as a clarification: Volubility (protophones per min) as determined from Phase 1 was not the only criterion for selection of the 8 Phase 2 segments, We also selected for infant directed speech (IDS), based on a Phase 1 questionnaire item (for four of the eight), as well as for segments where infants were deemed to have likely been alone and not engaged in vocal interaction (low IDS) (for two of the eight). The remainder were selected for volubility only. We have added a Supplementary item that consists of the detailed description of the Phase 2 segment selection algorithm. One of the key outcomes of the Phase 2 selection procedure was almost total elimination of segments where the infants were deemed to be asleep in Phase 2. Other outcomes were relatively higher volubility and relatively higher IDS than in randomly selected segments.**

**The average volubility was indeed higher in the 8 selected segments per recording (7.4 prot/min) than in the 21 randomly selected ones (sleep segments were not included in the calculation) (4.9 prot/min).**

**As for the hypothesis that volubility might positively predict VP, the result surprisingly showed a weak (and not statistically significant) *negative* correlation between volubility and VP (segments with some VP as opposed to no VP, *ρ* = -.022). For the 5 point scale of VP with volubility the negative correlation was stronger and was statistically significant (*ρ* = -.122, *p < .001*). As we understand the reviewer’s implication, if VP were predicted positively by volubility, but TT were not, then the greater rate of VP relative to TT might be overestimated in our analysis (with respect to a possible outcome based on random selection of segments, where overall volubility is lower). But actually it was TT (some TT vs. no TT) that was positively (and statistically significantly) correlated with volubility (*ρ* = .132, *p < .001*), and was even more strongly correlated with volubility for the 5 point scale on TT (*ρ* = .151, *p < .001*). If we do not misunderstand the reviewer’s suggestion, it would seem that if anything, our sample might be thought to have *underestimated* the difference between rates of VP and TT. In the revision, we have added a paragraph indicating that the relatively high volubility of the selected segments did not appear to have biased the sample toward more VP than TT.**

**Another factor that suggests we may have minimized the difference between VP and TT with our Phase 2 segment selection procedure is that, by definition, TT can only occur when a caregiver is vocally engaged with the infant (i.e., producing IDS). Consequently there was relatively higher IDS (based on the 5 point scale for IDS in the Phase 1 questionnaire) in the Phase 2 segments (mean = 2.13) than in the segments that were NOT selected for Phase 2 (mean = 2.04). The correlation between TT and IDS within the selected segments was positive and highly significant (*r* = .41, *ρ* < .0001), suggesting that the bias in selection toward higher IDS may have minimized the difference found between VP and TT.**

**On the other hand there are a variety of additional potentially important variables to consider that may interact with volubility and its relation with VP and TT in ways that might with continuing research require revision to speculations about other influences. One of the main reasons for our seeking to publish this paper promptly is that the difference between rates of VP and TT was so massive as to constitute what we have called a “landscape effect”, that is, a pattern so salient that it seems impossible any additional variable could overturn the apparent finding. The differences were greater than any of us anticipated, and we interpret them as having important implications both theoretically and practically.**

This could also affect proposition 2, as I would hypothesize that there is more canonical babbling in highly voluble bouts of VP than low-volubility VP. Perhaps I am misunderstanding something important about this selection that the authors can correct or justify, but as it stands I would really like to see some data speaking to this issue to justify the conclusions.

**We agree that this is a potentially important matter. But we have no measure of CBR in the Phase 1 (randomly selected) segments. Consequently, we have no way to assess the relation between CBR and volubility in randomly selected segments based on the available data. Assessment of the reviewer’s potential objection, as we think we understand it, is confounded by the massive imbalances in the numbers of segments showing some VP vs no VP (>90% some VP) and between the numbers of segments showing some TT vs no TT (80% no TT).**

**We can confirm that (as suggested by the reviewer) if we implement a median split of the data based on volubility, and then compute the mean CBR for segments with some VP for segments above the median for volubility, the mean is higher than for segments below the median (.117 vs .040). The same is true of the mean CBR for segments with some TT: for segments above the median volubility CBR was higher than for segments below the median (.159 vs .099). This outcome is consistent with our interpretation that infants may have been naturally selected to produce their most advanced vocalizations during particularly active periods—periods of high VP, high TT and high volubility can all be viewed as periods of high vocal activity.**

**At the same time, volubility was not as highly correlated with CBR as either VP or TT: volubility with CBR, *r* = .090; VP (some vs none) with CBR, *ρ* = .160; TT (some vs none) with CBR, *ρ* = .196; VP (5 point scale) with CBR, *ρ* = .167; and TT (5 point scale) with CBR, *ρ* = .215. Interestingly, as indicated above, volubility correlates negatively in our sample with VP (*ρ* = -.022 for some vs no VP and *ρ* = -.122 for the VP 5 point scale), while volubility correlates positively with TT (*ρ* = .132 for some vs no VP and *ρ* = .151 for the TT 5 point scale).**

**Reinforcing a general point: This was not an experiment, and there are a variety of possible factors that may have been involved in driving the CBR outcomes. The aforementioned massive imbalances (in opposite directions) for numbers of segments with or without VP or TT may make it difficult to sort out the role of volubility and a variety of other possible parameters in the outcomes for CBR. In response to the reviewer’s apparent concern that selecting segments with relatively high volubility from among the random ones could somehow skew the data on CBR with regard to VP or TT such that the outcome would have been different if we had coded all the randomly selected segments, we do not see how the data we have can rule out that possibility. In a future study, perhaps we can compare CBR with regard to VP and TT in randomly selected segments.**

Coder agreement
More needs to be done to justify the use of the VP/TT categorizations with agreement this low. I appreciate the data point the authors provide that conclusions around the higher proportion of VP than TT hold regardless of the set of ratings used, despite low agreement. However, I find it a little disingenuous to refer to this as “the most important matter” and state that “the outcome conclusion would have been the same” when this is only 1 of 4 key hypotheses tested. The authors should explore whether the different sets of ratings would have an impact on the CBR conclusions, as well. Additionally since the authors dichotomize TT and VP for those analysis, I’d be more interested in this binary agreement than the correlation of the 1-6 ratings (I suspect it may look better).

**We did not intend to mislead the reader. The statements in question were actually explicit in indicating that the “most important matter” claim was specifically about the difference between VP and TT rates (the first proposition). It made no reference to any of the CBR propositions, for which we have insufficient agreement data to support a similar outcome comparison. We see, however, that the phrasing left open an interpretation we did not intend. We have eliminated the “most important matter” statement and reworded to make it even more clear that it pertains only to the VP vs TT comparison. In the revision we merely point out that there is another way (beyond correlations) to view agreement for the VP vs. TT comparison, and it supports the conclusion that the difference would have been found even if we had used different coders.**

**For the other propositions, 2-4, the agreement data corresponded to fairly high correlations among coders: r = .872 in the case of the 105 segments data with 7 agreement coders, and .714 in the case of the 60 segments data with 4 agreement coders (in both cases, p < .001), as was reported in the prior text.**

**However, the magnitude of agreement correlations on a measured parameter is not required to be high in order for agreement to be sufficient to justify statistical comparisons of groups on that parameter as long as the correlations themselves are statistically significant, which of course all these correlations were at very high levels (even the ones on VP and TT). Asystematic coder disagreement creates noise in any group comparison, and consequently the danger is not Type I, but Type II error (1). Since we obtained statistically significant correlations among coders for all the comparisons (propositions 1-4), it seems reasonable to conclude that whatever the coder disagreement level was, the noise it created was over-ridden by the magnitude of the real differences. We have added a paragraph along with a relevant citation (1) on this point in the section on coder agreement.**

Results
In general the switching between Cohen’s d and mean difference was a little hard for me to track in this whole section. For example the sentence: “Another perspective on the size of the mean difference (.092), however, is that it exceeded the well-known growth in CBR by Age—based on the present data, the CBR growth from 7.5 to 12 months was .081.” was inscrutable to me upon first read. Clarifying that the mean difference here is the difference in CBR between Some VP and No VP would be helpful.

**Done.**

Limitations

It would be nice to acknowledge the lack of video ground truth for coding VP and TT. It seems probable that in many instances of VP, infants are looking at a caregiver as they do it, suggesting a social role even if TT does not occur, which would render VP not strictly endogenous.

**We agree that video plus audio would be preferable (always) and hopefully in the not too distant future, all-day recordings with video will be practicable. In response to the reviewer’s concern, we have added a statement in the Audio Recordings section, indicating that audio/video recordings are desired for the future of such research.**

**Our findings, however, are strengthened by the fact that we *have* evaluated in-laboratory, short-term recordings, that do involve both video and audio. The most telling of these may be the one published in PLoS One in 2020 (2), showing that *even in a circumstance where parents were instructed to interact with their infants, fewer than half the protophones of the infants included parent-directed gaze or any other pattern of vocalization that suggested directivity of the protophones to a person*. The gaze direction pattern was supported by earlier coding of video plus audio with a separate group of observers (3), Supplementary Material. These observations do not rule out the possibility that some of the vocalizations that coders judged as VP based on the audio recordings could have actually constituted TT, but the opposite is also true—especially in segments where multiple individuals were talking simultaneously, it often seemed likely that utterances of infants could have been falsely interpreted as TT. Subsequent research comparing audio/video coding with audio only coding will be needed of course to sort out these possibilities.**

Reviewer #3: I was not a reviewer of the original ms, so my comments pertain only to the revised submission. However, I did read the reviews and noted the very extensive changes to the current ms. I can see that the original reviews were highly effective and that the new ms is substantially improved, with a clearer focus as well as more complete results in relation to the issues raised.
I find the basic empirical findings highly convincing – that vocal play is far more frequent than turn-taking, that both types of vocal activity increase over the age range of the study and that canonical babbling is more characteristic of turn-taking than of simple vocal play, despite the fact that the former is far less common. In general, it is apparent that the study – selection of ‘segments’, coding, reliability checks, statistical analysis – was very carefully conducted; I will have nothing to say about those aspects. Instead, I will focus on the abstract, introduction and conclusion and related literature.

Although the abstract seems to be correct as far as it goes and is perfectly clear, a very large proportion of the non-data-based portions of the paper are directed at evolutionary theory. These aspects of the Introduction and Discussion are interesting and worth highlighting – yet there is no mention of them whatsoever in the abstract. This seems to me to be an avoidable error.

**Thanks for the suggestion and the encouragement. We have revised the abstract to include the evolutionary perspective.**

I find the literature review somewhat incomplete and the main claim that the paper is designed to challenge – that vocal activity in this period is largely based on imitation, or more precisely, that ‘phonological forms are learned in the first year by infant imitation’ (53f.) – to be something of a straw man (see also 85f, 750ff. and later). More specifically, in a chapter on Precursors to language in Phonological Development (2014, 2nd ed.) Vihman, based on considerable tapping of the literature, presents a view that is not unlike what this paper is meant to show (ll. 755ff.: ‘the possibility that most of the progress in speech-like vocalization of infants that occurs across the first year is the product of…exploratory activity’).

**Thanks for the many thoughtful comments.**

**Good point about the literature review. We should have noticed the reviewer’s point in the prior version of the ms. The relevant literature is split into 1) a very large infant/child development domain of research (with hundreds of relevant citations) that has very much focused on parent-infant vocal interaction and on infant vocal imitation; endogenous vocalization is not explicitly ruled out in this literature, but it receives scarcely any attention; as opposed to 2) a much smaller (but we think, along with the reviewer) more well-informed domain of research that recognizes endogenous vocal activity of infants, and in several cases, including those cited in Vihman’s work, that have long expressed skepticism about the standard child development expectations. We have revised the introduction to take account of the existence of a long-standing (rather than only recent) trend of scholarship expressing the empirically based conviction that infant vocalization is commonly if not predominantly endogenous.**

**We have also tried to make clear that although there has long existed published speculation about the importance of endogenous vocalization in infancy, *we know of no prior attempt to quantify the imbalance in rates of VP vs TT*, *or for that matter, any attempt until very recently to quantify endogenous vocalization in infancy at all.* We were amazed by the magnitude of the difference between VP and TT obtained in the present work, even though a notable proportion of the papers that have argued for the importance of endogenous infant vocalization have come from our own laboratories and those of our collaborators. The primary theoretical claim that exploratory vocalization is central in development was published as early as 1981 (4).**

And again, reference to Locke’s important 1993 book is good, but one would expect to also see a reference to Vihman et al., 1985, which demonstrated empirically the relationship of infant babbling to their word forms.

**Done.**

More importantly, however, the paper’s evolutionary bias sometimes seems to lead the authors to disregard alternative interpretations. For example, we are told that Higher rates of canonical syllables during social interaction than during periods without social directivity would suggest a social motivation for producing more advanced protophones. If the idea is on target, we might propose that canonical babbling has been specifically selected to provide a salient signal of developmental progress, especially during social interaction. (ll. 178-181, italics added)

Alternatively, might it not be that infants are stimulated or ‘primed’ by hearing IDS, once canonical babbling is in repertoire, to produce their more advanced, more adult-like babble? Need we jump to the conclusion that they are ‘signalling’ something at this stage (cf. l. 245), when there is little evidence of intentional vocal communication (which tends to emerge only at 13 mos. or so: McCune et al., 1996)? The authors hint at something like this themselves, ll. 490f.

**Pardon us if we write too much here, but we are unsure where misunderstandings or disagreements may exist. We’ll cover enough ground to account for several possibilities.**

**First, let us refer to Tinbergen’s four questions (5): As we understand it, the reviewer is referring to “alternative interpretations” that pertain to Tinbergen’s “proximate” questions about why a behavior (in this case higher rates of CB in some cases than others) occurs from the standpoint of 1) causality and 2) development, while our paper focuses on Tinbergen’s “ultimate” questions concerning 3) survival value and 4) evolution. Of course, the proximate questions are always of interest, and so we have made adjustments in the revised text in several places to more prominently acknowledge their interest and importance along with citations the reviewer will hopefully find appropriate.**

**On the other hand, unless we misunderstand the reviewer, we think it may be worthwhile to expand upon our use of the terms “signal” and “intention,” where it appears either our opinions or our definitions (or both) are different from those of the reviewer.**

**Our claim that fitness signaling is essential to the origin of protophone production (including canonical babbling, which is a kind of protophone) implies that, at least sometimes, there exists a social interaction motive in infancy to produce protophones in addition to the motive to explore protophones in independent vocal activity. We view the social motive as having been naturally selected (in Tinbergen’s ultimate sense) through essentially the same mechanism as the motive to produce protophones in independent vocal play; the primary ultimate mechanism in both cases was, according to our proposal, selection for signals of fitness. Having seen the tendency for CBR to be especially high during periods of VP and TT (and highest of all during TT), we were struck by the seemingly obvious implication that the natural selection pressure on fitness signaling by vocalization logically should have been expected to produce infants who not only explore vocalization in ways that *might be heard* by caregivers, but also that they should be expected to vocalize in ways that *maximize their fitness signaling value*, that is by producing the most advanced of their vocal products at those points in time that caregivers are most likely to notice them. This does not suggest a change in “priorities” as the infant becomes more developmentally advanced, but simply suggests that the same inclination to signal fitness is implemented with more advanced materials as the infant becomes more vocally capable.**

**It appears we may be using the term “signal” in a way that the reviewer finds unusual or inappropriate. In fact the theory of the origin of infant vocal exploration requires that the protophones be signals from the very beginning of life. It seems necessary here to explain why, both theoretically and empirically, we reject the idea that intentional vocal communication does not pertain to the first year. On the contrary, we believe it is clear that there exists intentional vocal communication by the human infant even within the first few months.**

**But let us step back and explain first why protophones constitute “signals” from the beginning of life, even if they are *not,* from the infant perspective, intentionally communicative. During periods of obvious vocal play, our interpretation is that the “intention” in vocalizing is exploration itself. Such vocal play vocalizations can serve as fitness signals because, in accord with the fitness signaling hypothesis, caregivers must sometimes notice those vocalizations and interpret them as indicators of infant state even when they are not looking at the infant and even if the infant does not intend for the vocalizations to be heard. If it were not so, the fitness signaling hypothesis would be greatly weakened, because it is the hominin caregiver who is posited to have supplied the selection force on generation-by-generation growth in the infant tendency to produce reliable signals of their wellness through vocalization. We reason that caregivers selectively invested in infants who showed the inclination to vocalize in a way that displayed wellness and that such selection led to infants finding vocal exploration endogenously rewarding. Notice that because caregivers can interpret vocal play as a fitness signal, it may not be intentionally communicative, but it succeeds in transmitting communication about fitness nonetheless, in our interpretation.**

**In face-to-face vocal interaction in the first month of life, it seems reasonable to be skeptical (as apparently the reviewer is) about whether infants “intend” to signal anything by apparent vocal responsivity. It’s not clear, for example, how to rule out the possibility that an infant could be acting in the first month like a coupled oscillator in a purely physical system, responding to the rhythmicity of the parental vocalizations with a somewhat similar rhythmic vocal pattern of their own. Yet again, however, it is not necessary for the infant to *intend* to signal wellness in order for the infant vocal action to constitute a wellness signal from the caregiver perspective. Even if the caregiver never thinks about it consciously, a vocally responsive infant shows an ability to respond to vocal contingency which may be tacitly read by the caregiver as a sign of wellness.**

**The distinction between what the infant intends and what the caregiver may interpret on the basis of the infant action needs always to be maintained in order to recognize the sense in which our proposal avoids the untenable suggestion that the natural selection force supporting infant vocal activity at an early stage of development or evolution could have been based on an infant need for or desire for language or an intention to develop language—language did not yet exist, in accord with the hypothesis, for the ancient hominins who first engaged in endogenous, exploratory vocalization, and we contend that it is also unreasonable to suggest the modern infant’s tendency to vocalize in interaction during the first months is motivated by an attempt to develop language in the mature sense of the term. Ancient hominin caregivers had to supply the selection pressure on infants who revealed their fitness by exploratory and/or interactive vocalization. Ancient hominin infants did not intend to develop language, but they may have intended to display their fitness, especially during vocal interaction.**

**As infants progress beyond the first two months, the evidence mounts that infants in face-to-face interaction are systematically engaged in vocal interchange, showing ever stronger signs of contingent interaction, and thus intentional communication, than at earlier ages. Especially after 5 months of age, there is solid evidence that modern human infants systematically engage in vocalization that appears to be directed to signaling toward the parent, vocalizing in a manner that seems intended to re-initiate a broken interaction. The most salient results come from studies in the Still-face paradigm, where infants after 5 months, double (or more than double) their vocalization rates when parents shift from the Interactive phase to the Still-face phase (6-8). At 3 months vocal rates remain essentially unchanged at the Still-face shift (8). This pattern has been interpreted as an indication of infant vocal communicative adaptation by 5 mo, and it is hard to see how the result can be interpreted as anything less than an attempt by the infant to vocally re-engage the suddenly inert parent. As the year progresses for infants, there are increasingly clear indicators (even if relatively infrequent in occurrence) that infants are gaining the capacity to communicate intentionally with vocalization (for example, the very infrequent cases of immediate vocal imitation and the obvious appearance of protowords in many infants, of which the reviewer is obviously fully aware).**

**While the described infant vocal activity in these first-year interactions is not language (except perhaps in instances where infants learn to use a few words), it is a foundation for language in the sense that language is inherently socially interactive, and it is communicative in the sense that it proves to the listener that the infant intends to vocally interact. On this point we take issue with McCune et al. (9), whose view of the notion “intentional communication” is, we think, overly restrictive, and seems to have applied primarily if not exclusively to the vocalizations they called “grunts”, about which more below.**

**The difference of opinion could also be predominantly one of how the term “intention” is interpreted. Our interpretation of the term (that infant protophones, even in vocal play are intentionally exploratory) is critical to the fitness signaling hypothesis.**

**On the suggested “alternative” to the fitness signaling hypothesis that infants might be “stimulated or ‘primed’ by hearing IDS, once canonical babbling is in repertoire, to produce their more advanced, more adult-like babble…” we agree that this suggestion represents a “proximal” description of infant adaptation, and we have added to the text to acknowledge that agreement. However, the description does not provide an “ultimate” mechanism by which that adaptation could have emerged in a non-linguistic world. Our contention is that the fitness signaling idea still needs to be invoked to explain how the infant could have come to have the exploratory and social interactive motivations that led to the proximal adaptations.**

And again, in discussing the unanticipated finding of increases in both vocal play and turn-taking between 7 and 12 months the authors say, “It makes sense to suggest that infants ramp up their vocal activity in all circumstances as time passes, as the possibility of genuine linguistic communication approaches, and as the priority on learning both through exploration and through interaction grows”.
But of course, as the authors are at pains to say themselves, infants (like evolution) cannot anticipate what will be of use later, so that the ‘approach’ of genuine linguistic communication can’t be causal, though it may be caused by this ramping up. Maybe it isn’t infants’ priorities that are changing over this time but rather their mastery of consonant production, their increased supply of well-practiced vocal forms, including syllables, and their sensitivity to a match between the forms they produce and the forms they hear in adult talk, especially IDS, presumably. This is the basis for Vihman’s notion of an ‘articulatory filter’ (1993), a view that the authors appear to echo on ll. 219ff: ‘there may be a tendency of infants to explore their most speech-like utterance capabilities when they are most actively involved in VP, times during which they may be most attentive to their own vocal product, stimulating awareness of their category growth and calibrating their vocal capacities’.(italics added). Most recently, Vihman has suggested that babble functions to create phonological memory, a launchpad for word formation (2022). It would be useful to include such alternative interpretations in the discussion.

**About the notion of shifting “priorities,” see above. As for the possibility that the infant at some point (especially in the second half year) may begin to engage in some interaction that involves actual (and intentional) early language learning, we think we are in agreement (if we understand the reviewer’s point) that such intentional communication and learning begins to occur during that period. We have also added a citation to the articulatory filter idea as an important theory and proximal characterization of how infant phonology begins to emerge with babbling as a foundation.**

**Our view is that if substantial vocal exploratory activity had not occurred early in life, along with vocal interaction where contingency between caregiver and infant could have been both explored and developed, infants would not have established the foundations necessary to take additional steps at a later stage such as attending to parental vocalizations in terms of their phonetic content during face-to-face interaction. Notice that even in the circumstance where infants actually begin to learn something substantive about pronunciation of words and sounds *from* caregivers, infants should still be viewed as engaged in fitness signaling (in addition to their focused attention on words and sounds of caregivers). This step by step viewpoint is presented (with many citations to existing empirical evidence) as an infrastructural natural logic that outlines steps required for vocal language to be built, with exploratory vocalization constituting a necessary very early step (10, 11). The natural logic is designed in such a way that it has plenty of room to accommodate the articulatory filter as a key development to the extent that we understand the idea.**

Some more specific comments:
…‘babbling’ is very often used as a short form of ‘canonical babbling’, although it is sometimes used to cover infant vocalizations of the kind Oller and his colleagues term ‘protophones’. This is a systematic source of confusion in the literature and the parenthetical ‘roughly “babbling”’ on l. 76 does not seem helpful (see, for example, the very different use of the same term in McGillion et al., 2017).

**Yes, this is a longstanding terminological problem. The term “protophones” was coined (11) specifically to try to provide a solution to the problem—it was designed (and Oller and colleagues have always used it this way) to refer to both noncanonical and canonical speech-like vocalizations of infants, thus presumably supplanting the common parlance usage “babbling” as it has appeared so often in the literature to refer on some occasions 1) to both precanonical and canonical vocalizations, and on other occasions 2) to canonical babbling only. The reviewer is not alone in not having understood the term protophone the way we have always intended it—we have encountered the same confusion among both primatologists and developmental psychologists who are familiar with our work. We have been trying to correct that confusion every time we write about the protophones. So we have revised the section in question to try to be more explicit that the term protophones is intended to encompass both noncanonical and canonical infant speech-like utterances.**

The authors reference ‘effort grunts’ but do not tell us what they are. Note that in this period ‘attention grunts’ have also been observed, although ‘communicative grunts’ appear to arise only beyond age 12 mos.: see McCune et al., 1996, Grünloh et al., 2015. Are these included under protophones or not? They may be the vocal forms that facilitate production of such early-produced word forms as oh.

**In the revision we define more explicitly and attempt to clarify that the term “grunts” as we use it. For us, the term always refers to effort grunts, which are** **required to have a sharp glottal onset and to accompany (and in a sense be artifacts of) some kind of movement or straining that seems to required a glottal hold to impose rigidity on the internal torso—the effort grunt occurs at the release of the glottal hold.**

**The reviewer may be interested in some additional commentary on the matter of “grunts”, so we continue here for the reviewer’s interest.**

**The utterances McCune et al. (9) call “attention” grunts and “communicative” grunts, would never be referred to as grunts in our work, although this is primarily a terminological issue. They would always instead be called “quasivowels”, as far as we can tell, although for real-time coding in the OLL, quasivowels are a subcategory of “vocants” or vowel-like sounds.**

**Quasivowels begin to occur on the very first day of life (and at least in the first month in our longitudinal studies that include infants that young), and account for the nuclei of over 40% of protophones at 4 months (12), about 30% of all phonated syllables even as late as 6 months, 20% at 8 months and a little under 20% thereafter through 18 months (13). They are often auditorily similar to effort grunts (although normally distinguishable) to the extent that they involve phonation with a supraglottal tract at rest. For a quasivowel, the tract can be closed or very slightly open, but not postured as if for any particular vowel, but quasivowels differ from effort grunts (which often have very similar supraglottal posturing) in that quasivowels: 1) are not required to include (and usually do not include) a sharp glottal onset, 2) cannot exhibit signs of being movement artifacts, 3) can be considerably longer than effort grunts usually are, 4) are often included as nuclei in syllables (a type of “marginal syllables” in our usage) that may include one or more consonant-like closures as well as a quasivowel nucleus, and 5) are much more likely than effort grunts to involve normal phonation only (whereas effort grunts often include harsh phonation). In our own work we take it to be important to have a term to refer to quasivowels, because we see a gradation in resonances from quasivowels to the vowel-like sounds that involve a postured vocal tract and that are the hallmark of nuclei in the vast majority of syllables in mature natural languages.**

**McCune et al. (9) have reported that it is only (or largely) in the second year that “grunts” (their term) are used “communicatively”. This is an interesting finding. We wonder if McCune et al. were influenced in their coding to refer to acts as communicative especially (or exclusively?) if they occurred in triadic communication: e.g., Mom says, pointing, “that’s a horse”, and baby responds with a quasivowel, with the illocutionary force of agreement or assent about the lexical reference. Or Mom says “let’s put you in your high chair” and the baby responds with quasivowel assent, having understood the reference to either the high chair or the proposed action or both. We adults indicate acknowledgment, agreement, assent, encouragement for a speaker to continue, sometimes disapproval, and so on with quasivowels also, and in the common parlance these quasivowels are often called grunts (as McCune et al. point out). “Tom was asked what he thought, and he grunted to indicate his agreement with the previous comment.”**

**As for “attention grunts” (again McCune et al.’s usage), we suspect that these would not only be called “quasivowels” in our approach, but that they would usually qualify as vocal play, since we treat protophones not directed to a person and not constituting whining, as exploratory vocalization (perhaps it should be noted that whining can accompany quasivowels). Quasivowels occur in a wide variety of circumstances, but it had never occurred to us until reading McCune et al. that perhaps they are not used with eye-contact until the second year. We’ll take note of this possibility as we continue our research in both the first and second year.**

…use of present perfect where past tense would be appropriate: l. 190, l. 260

**Corrected.**

**References to the responses in the letter:**

1.	Hallgren KA. Computing Inter-Rater Reliability for Observational Data: An Overview and Tutorial. Tutorials in quantitative methods for psychology. 2012;8(1):23-34.

2.	Long HL, Bowman DD, Yoo H, Burkhardt-Reed MM, Bene ER, Oller DK. Social and endogenous infant vocalizations. PLOS ONE. 2020;15(8):e0224956.

3.	Oller DK, Buder EH, Ramsdell HL, Warlaumont AS, Chorna L, Bakeman R. Functional flexibility of infant vocalization and the emergence of language. Proceedings of the National Academy of Sciences. 2013;110(16):6318-632.

4.	Oller DK. Infant vocalizations: Exploration and reflexivity. In: R.E.Stark, editor. Language behavior in infancy and early childhood. New York: Elsevier North Holland; 1981. p. 85-104.

5.	Tinbergen N. On aims and methods of ethology. Zeitschrift für tierpsychologie. 1963;20(4):410-33.

6.	Goldstein MH, Schwade JA, Bornstein MH. The value of vocalizing: Five-month-old infants associate their own noncry vocalizations with responses from adults. Child Development. 2009;80(3):636 – 44.

7.	Franklin BS, Warlaumont AS, Messinger DS, Bene ER, Iyer SN, Lee C-C, et al. Effects of parental interaction on infant vocalization rate, variability and vocal type. Language Learning and Development. 2014:1-18.

8.	Yale ME, Messinger DlS, Cobo-Lewis AB, Oller DK, Eilers RE. An event-based analysis of the coordination of early infant vocalizations and facial actions. Developmental Psychology. 1999;35(2):505-13.

9.	McCune L, Vihman MM, Roug-Hellichius L, Delery DB, Gogate L. Grunt communication in human infants (Homo sapiens). J Comp Psychol. 1996;110(1):27-36.

10.	Oller DK, Griebel U, Warlaumont AS. Vocal development as a guide to modeling the evolution of language Topics in Cognitive Science (topiCS), Special Issue: New Frontiers in Language Evolution and Development, Editor, Wayne D Gray, Special Issue Editors, D Kimbrough Oller, Rick Dale, and Ulrike Griebel. 2016;8(2):382-92.

11.	Oller DK. The Emergence of the Speech Capacity. Mahwah, NJ: Lawrence Erlbaum Associates; 2000. 428 p.

12.	Steffens ML, Oller DK, Lynch MP, Urbano R. Vocal development in infants with Down syndrome and infants who are developing normally. American Journal of Mental Retardation. 1992;97:235-46.

13.	Oller DK, Eilers RE, Steffens ML, Lynch MP, Urbano R. Speech-like vocalizations in infancy: an evaluation of potential risk factors. Journal of Child Language. 1994;21:33-58.
