# Supplementary material for: Perspectives on the origin of language: Infants vocalize most during independent vocal play but produce their most speech-like vocalizations during turn taking
Source: PLoS One. 2022 Dec 30;17(12):e0279395. doi: 10.1371/journal.pone.0279395 (PMC9803194; doi:10.1371/journal.pone.0279395)
Supplement: Attachment — Submitted filename: Response to Reviewers2.docx [file pone.0279395.s010.docx]

Response letter for PONE-D-22-03801R3

Perspectives on the origin of language: Infants vocalize most during independent vocal play but produce their most speech-like vocalizations during turn taking

Dear Editor (Professor Perlman):

**Our responses to reviewer comments are in boldface below.**

Reviewer #2: Thank you for the opportunity to review this second revision of the manuscript. The authors have done extensive work to address my concerns, and I am satisfied with the revisions. I have 2 minor comments:

Last sentence of abstract: “The results inform our proposal that the human infant has been naturally selected to explore protophone production and that the exploratory inclination formed a foundation in our hominin ancestors without which language could not have evolved.” The phrase “without which language could not have evolved” seems excessively strong and is not shown by this work. I am in favor of removing it.

**Thank you for this suggestion. We have revised the last sentence of the abstract to the following: “The results inform our proposal that the human infant has been naturally selected to explore protophone production and that the exploratory inclination formed a foundation in our hominin ancestors without which language could not have evolved.”**

Line 375-376: “Volubility could then be thought to drive a tendency for high CBR in TT if the results show that pattern.” I’m not actually sure what “if the results show that pattern” means. Does it mean “if canonical babbling is indeed associated with volubility”? It would be nice to clarify this.

**We have clarified this sentence at lines 375-376 to the following: “Volubility could then be thought to drive a tendency for high CBR in TT if the results show that pattern.”**
